# Regenerable and stable *sp*^2^ carbon-conjugated covalent organic frameworks for selective detection and extraction of uranium

**DOI:** 10.1038/s41467-020-14289-x

**Published:** 2020-01-23

**Authors:** Wei-Rong Cui, Cheng-Rong Zhang, Wei Jiang, Fang-Fang Li, Ru-Ping Liang, Juewen Liu, Jian-Ding Qiu

**Affiliations:** 1 College of Chemistry, Nanchang University, Nanchang, 330031 China; 2 Department of Chemistry, Waterloo Institute for Nanotechnology, University of Waterloo, Waterloo, Ontario, N2L 3G1 Canada

**Keywords:** Polymers, Sensors and biosensors, Polymers

## Abstract

Uranium is a key element in the nuclear industry, but its unintended leakage has caused health and environmental concerns. Here we report a *sp*^2^ carbon-conjugated fluorescent covalent organic framework (COF) named TFPT-BTAN-AO with excellent chemical, thermal and radiation stability is synthesized by integrating triazine-based building blocks with amidoxime-substituted linkers. TFPT-BTAN-AO shows an exceptional UO_2_^2+^ adsorption capacity of 427 mg g^−1^ attributable to the abundant selective uranium-binding groups on the highly accessible pore walls of open 1D channels. In addition, it has an ultra-fast response time (2 s) and an ultra-low detection limit of 6.7 nM UO_2_^2+^ suitable for on-site and real-time monitoring of UO_2_^2+^, allowing not only extraction but also monitoring the quality of the extracted water. This study demonstrates great potential of fluorescent COFs for radionuclide detection and extraction. By rational designing target ligands, this strategy can be extended to the detection and extraction of other contaminants.

## Abstract (web summary)

Porous materials for uranium capture have been developed in the past, but materials for simultaneous uranium capture and detection are scarce. Here the authors develop a stable covalent organic framework capable of adsorbing and detecting uranyl ions.

## Introduction

With a low-carbon footprint, nuclear energy has a critical role in the global energy system^1–3^. Owing to the widespread use of nuclear power, large-scale uranium mining, nuclear accidents, and improper disposal of nuclear wastes, a large quantity of radioactive uranium has penetrated into the environment mainly in the form of UO_2_^2+^ ^4–6^. Thus, regenerable materials for concurrent UO_2_^2+^ detection and extraction are demanded for environmental monitoring and protection.

Some porous materials such as porous organic polymers (POPs)^7^, metal-organic frameworks (MOFs)^8^, and hydrogels^4^ have been developed for this purpose. However, the performance of amorphous POPs is affected by its irregular pores, burying a large fraction of porosity^9^, and hindering fast mass transfer needed for real-time response^10^. Although MOFs have regular pores and good crystallinity^8^, stability under extreme conditions (acid, base, temperature, and radiation) remains a challenge^11–13^. High stability is particularly important for the extraction of UO_2_^2+^, as the sample matrix is likely to be strongly radioactive and acidic. Therefore, it remains a synthetic challenge for real-time detection and regenerable extraction of UO_2_^2+^.

Covalent organic frameworks (COFs) are a class of porous crystalline polymers with significant advantages for application in catalysis^14–18^, gas storage^19–22^, and metal ion extraction^23–27^ owing to excellent chemical and thermal stability, flexible topological connectivity, and tunable functionality^28–30^. COFs with tunable porosity and large specific surface area might be ideal for extracting radionuclides such as UO_2_^2+^. In addition, post-modification can rationally place various functional units within the periodic arrays to optimize the performance. At present, various COFs based on the Schiff base reaction (for example, COF-TpAb-AO^1^, and *o*-TDCOF^3^) have been developed for the extraction of UO_2_^2+^. However, their major covalent bonds, such as the boron–oxygen and imine bonds, are susceptible to irradiation, acid, and base, which greatly limit their regeneration and practical application^3,31,32^.

Recently, considerable attention has been paid to the construction of olefin-based COFs synthesized by the Knoevenagel condensation reaction. Although the *sp*^2^-carbon bond are very stable, the reversibility of *sp*^2^-carbon bond formation is poor, making the synthesis of *sp*^2^-carbon-linked COFs extremely challenging^33^. Since 2016, several examples of *sp*^2^-carbon COFs have been reported, such as *sp*^2^c-COF^34^, TP-COF^35^, Por-*sp*^2^c-COF^33^, and g-C_34_N_6_-COF^36^. However, their application for the detection or extraction of UO_2_^2+^ has not been explored. More importantly, the exploration of COFs for fluorescence detection of UO_2_^2+^ is still in its infancy, and most UO_2_^2+^-sensing platforms are often hampered by poor selectivity and a long response time^37–40^.

We herein report a *sp*^2^ carbon-conjugated COF for simultaneous detection and extraction of UO_2_^2+^ by integrating triazine-based building blocks with amidoxime-substituted linkers. This *sp*^2^ carbon-conjugated COF not only has good luminescence yield, but also excellent chemical and thermal stability. Its selective binding of UO_2_^2+^ is obtained by introducing amidoxime functional groups as ligands in the open 1D channels. Its real-time fluorescence response to UO_2_^2+^ can be visually observed and recycling was also confirmed using reversible uranium binding.

## Results

### *sp*^2^ carbon-conjugated COF for reversible uranium binding

To achieve an acid, base, and radiation stable, strongly fluorescent and selective UO_2_^2+^-binding COF, we had the following materials design considerations. Most current COFs relied on the boron–oxygen and imine bonds^35^. However, such reversible bonds lead to relatively poor stability. In addition, their weak π-electron delocalization over the framework hinders fluorescence yield^36^. Carbon–carbon double bonds (-C═C-) are more stable and can keep conjugated π-electrons, which can overcome the above challenges^36,41^. In addition, their intrinsic open 1D channels and regular porous structures facilitate exposure of binding sites, boosting rapid diffusion, and mass transfer. Thus, by introducing specific metal-binding sites on the pore walls, the *sp*^2^ carbon-conjugated COF may serve for high performance UO_2_^2+^ extraction under harsh conditions.

**Fig. 1:**
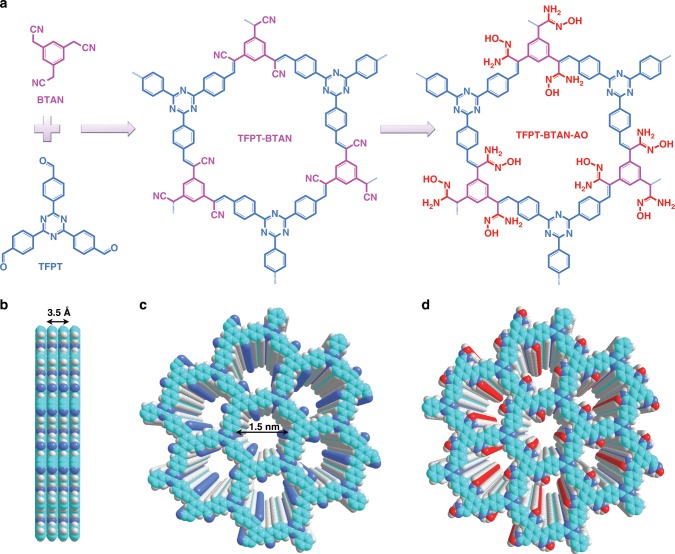
Schematic of synthetic TFPT-BTAN-AO. **a** Synthesis of TFPT-BTAN and TFPT-BTAN-AO. **b** Side and top **c** views of an eclipsed AA-stacking model of TFPT-BTAN (light green, C; blue, N; light gray, H). **d** Graphic view of TFPT-BTAN-AO (light green, C; red, O; blue, N; light gray, H).

Our synthesis is depicted in Fig. 1. 2,4,6-tris(4-formylphenyl)-1,3,5-triazine (TFPT) and 2,2′,2″-(benzene-1,3,5-triyl)triacetonitrile (BTAN) were polymerized through the Knoevenagel reaction, yielding a cyano-based COF (TFPT-BTAN). To overcome the low reversibility of the Knoevenagel reaction, we optimized the solvent, catalyst, and temperature (Supplementary Table 1 and Supplementary Fig. 1 in Supplementary Information), and highly crystalline TFPT-BTAN was prepared by condensing TFPT and BTAN in a mixture of *o*-DCB and 4 M DBU (10:1 by vol.) at 90 °C. Subsequently, TFPT-BTAN was subjected to amidoximation by treating it with an excess of NH_2_OH·HCl at 85 °C for 24 h to give TFPT-BTAN-AO. Our synthetic strategy not only constructed a highly stable π-conjugated skeleton as the fluorophore, but also introduced dense amidoxime groups as the uranium receptors. These unique features are expected to facilitate real-time detection and efficient extraction of UO_2_^2+^.

The chemical structure and composition of TFPT-BTAN were determined by Fourier transform infrared (FT-IR) and solid-state ^13^C CP/MAS NMR spectroscopy. In the FT-IR spectra of TFPT-BTAN (Supplementary Fig. 2), the characteristic vibration peak of -CN (at ~2241 cm^−1^) was observed for both BTAN monomer and TFPT-BTAN. A stretching vibration peak of C═O (at ~1698 cm^−1^) was found in the TFPT monomer and it completely disappeared in TFPT-BTAN, indicating a high degree of condensation. The solid-state ^13^C CP/MAS NMR of TFPT-BTAN further confirmed highly efficient condensation, supported by the peaks at ~113 and ~171 ppm assigned to the carbon atoms in cyano and triazine moieties, respectively (Supplementary Fig. 3).

**Fig. 2:**
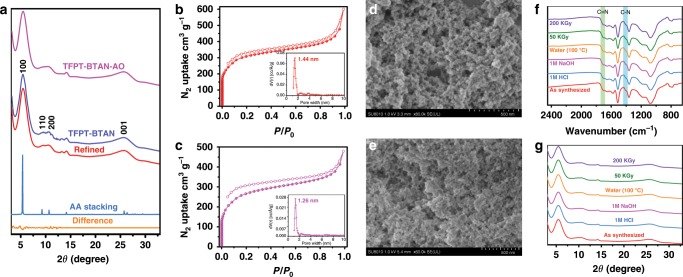
Characterization of TFPT-BTAN and TFPT-BTAN-AO. **a** PXRD profiles. Nitrogen adsorption–desorption isotherms of **b** TFPT-BTAN and **c** TFPT-BTAN-AO. Insets: the pore-size distributions calculated from non-local density functional theory. SEM images of **d** TFPT-BTAN and **e** TFPT-BTAN-AO. **f** FT-IR spectra and **g** PXRD profiles of TFPT-BTAN-AO before and after treatment with water (100 °C), HCl (1 M), NaOH (1 M), and γ-ray irradiation (50 kGy, 200 kGy).

To determine the structure of TFPT-BTAN, powder X-ray diffraction (PXRD) experiments were performed (Fig. 2a). A strong peak at 5.8° (2*θ*) is assigned to the diffraction from the (100) plane, indicating a highly crystalline of TFPT-BTAN^37^. The other peaks at 9.8°, 11.2°, and 26.3° (2*θ*) are assigned to the diffractions of (110), (200), and (001) planes, respectively. Our PXRD pattern matches well with the AA stacking model of the simulated TFPT-BTAN structure, and the Pawley refined PXRD pattern fits well with experimental data (*R*_p_ = 2.85% and *R*_wp_ = 4.27%), as demonstrated by the negligible difference (Supplementary Fig. 4 and Supplementary Table 2). The above results indicate that TFPT-BTAN has open 1D channels (1.5 nm in diameter) and the interlayer distance of the framework is 3.5 Å. To evaluate the details of the pore features of TFPT-BTAN, N_2_ adsorption–desorption experiments were performed. The Brunauer-Emmett-Teller (BET) surface area of TFPT-BTAN was determined to be 1062 m^2^ g^−1^, and the pore-size distribution centered at 1.44 nm based on non-local density functional theory, which matches well with the model (Fig. 2b)^30^.

We prepared TFPT-BTAN-AO by reacting crystalline and porous TFPT-BTAN with NH_2_OH·HCl. The PXRD pattern of TFPT-BTAN-AO shows a diffraction pattern comparable to the one of TFPT-BTAN with a strong diffraction peak at 5.8° (Fig. 2a), indicating that crystallinity was well retained after the amidoximation process. N_2_ adsorption–desorption isotherm were performed to verify pore accessibility after the post-modification process, affording isotherms comparable to those of TFPT-BTAN. The BET surface area was evaluated to be 803 m^2^ g^−1^, indicating that porosity was well retained after the amidoximation process (Fig. 2c). As shown in Supplementary Fig. 5, the vibration peak of -CN (at ~2241 cm^−1^) disappeared in TFPT-BTAN-AO, and the vibration peaks of the amidoxime groups can be observed at 1403 and 1707 cm^−1^, confirming the successful amidoximation^1^. Furthermore, solid-state ^13^C CP/MAS NMR spectra confirmed this successful conversion, as indicated by the disappearance of the peak at ~113 ppm that is assigned to carbon atoms in the cyano groups together with the concomitant appearance of a peak at ~156 ppm assigned to the carbon atoms in amidoxime groups (Supplementary Fig. 3)^1,35^. The obtained TFPT-BTAN and TFPT-BTAN-AO were pale-yellow powders. The scanning electron micrograph of TFPT-BTAN shows a porous network structure (Fig. 2d), which did not change after amidoxime functionalization (Fig. 2e), indicating that TFPT-BTAN-AO can be rapidly and thoroughly penetrated with UO_2_^2+^.

Thermogravimetric analysis profiles revealed that both the TFPT-BTAN and TFPT-BTAN-AO were stable up to 320 °C, indicating that good thermal stability (Supplementary Fig. 6). In addition, we treated TFPT-BTAN-AO under different harsh conditions for 12 h to study chemical stability. The FT-IR spectra (Fig. 2f and Supplementary Fig. 7a) and PXRD (Fig. 2g and Supplementary Fig. 7b) both show that TFPT-BTAN-AO retained the same characteristic peaks after the treatments, confirming its remarkable chemical stability. In order to reflect the superior stability of TFPT-BTAN-AO compared with other COFs, two *β*-ketoenamine COFs (Tp-Bpy and Tp-BD) were synthesized by the reported method^30^. After treatment with high concentrations of nitric acid (3.0 M and 5.0 M), the crystallinity of Tp-Bpy and Tp-BD was completely destroyed (Supplementary Fig. 7c and d), whereas our TFPT-BTAN-AO maintained good crystallinity and stability. The results show that TFPT-BTAN-AO has superior stability in high concentration nitric acid compared with *β*-ketoenamine COFs. Moreover, TFPT-BTAN-AO retained a high residual mass (≥93.5%) after treatment with different concentrations of nitric acid, further indicating its excellent stability (Supplementary Table 3). All of these indicated successful synthesis of stable TFPT-BTAN-AO.

### Selective sensing of UO_2_^2+^

After demonstrating the synthesis, we then studied the sensing performance of our COF. The normalized fluorescence spectra of TFPT-BTAN and TFPT-BTAN-AO dispersed in water are shown in Supplementary Fig. 8. Compared with TFPT-BTAN, the aminoximation process introduced a large amount of -OH and -NH_2_, thereby reducing the conjugation effect in the TFPT-BTAN-AO. As a result, the emission spectrum of TFPT-BTAN-AO blue shifted compared to TFPT-BTAN, which further indicates successful amidoximation. By excitation at 277 nm, TFPT-BTAN-AO emitted bright blue fluorescence at 460 nm, showing a high absolute fluorescence quantum yield of 4.3%. With a high-quantum yield and specific affinity uranium-binding groups, we then studied its UO_2_^2+^-sensing performance. A stock solution of TFPT-BTAN-AO was added to the solution containing UO_2_^2+^, and then diluted with ultrapure water for fluorescence measurement (Supplementary Fig. 9). The fluorescence of TFPT-BTAN-AO was significantly quenched by UO_2_^2+^, and the optimal pH for detection was at 6.0 (Supplementary Fig. 10). Importantly, TFPT-BTAN-AO responded very quickly to UO_2_^2+^, and the system can reach equilibrium within 2 s (Supplementary Fig. 11), much faster than other detection systems (Supplementary Table 4). These results suggest that the 1D channels could promote the rapid diffusion and mass transfer of UO_2_^2+^ and achieve real-time detection.

**Fig. 3:**
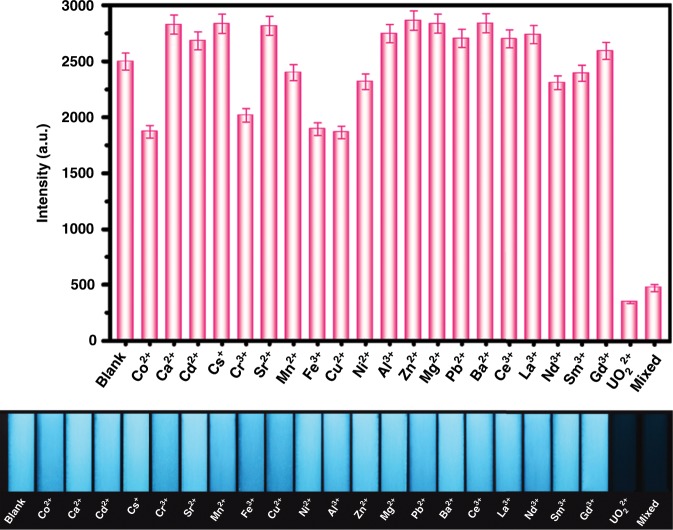
Selectivity investigations. Fluorescence intensity of TFPT-BTAN-AO at 460 nm in the presence of various metal ions and mixed ions. Concentrations of UO_2_^2+^ and other metal ions were 20 μM and 50 μM, respectively. Photographs showing the fluorescence emission change (under a portable 365 nm UV lamp) of TFPT-BTAN-AO with various metal ions. Error bars represent S.D. *n* = 3 independent experiments.

To test selectivity, UO_2_^2+^ (20 μM) was added directly to the TFPT-BTAN-AO aqueous dispersion, whereas other metal ions were added at 50 μM (Fig. 3 and Supplementary Fig. 12). Only UO_2_^2+^ caused significant fluorescence quenching, other metal ions have little effect on the UO_2_^2+^ detection, and it can be visually observed under a portable 365 nm UV lamp, indicating that TFPT-BTAN-AO has good selectivity for UO_2_^2+^ attributable to the specific affinity between UO_2_^2+^ and amidoxime groups^1^, and the fluorescence quenching property of UO_2_^2+^.

### Highly sensitive sensing of UO_2_^2+^

**Fig. 4:**
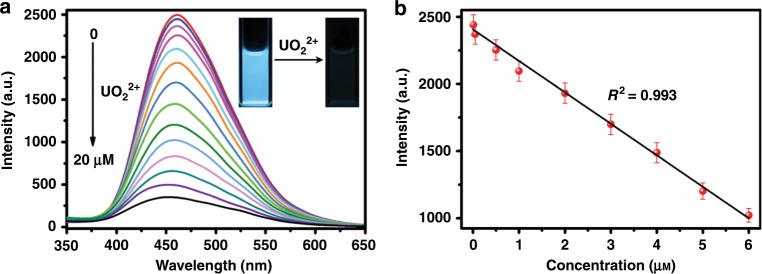
Sensitivity investigations. **a** Fluorescence emission spectra of TFPT-BTAN-AO upon gradual addition of UO_2_^2+^ (*λ*_ex_ = 277 nm). Inset photos show the fluorescence emission change (under a 365 nm UV lamp) of TFPT-BTAN-AO after addition of UO_2_^2+^. **b** Fluorescence intensity at 460 nm versus the concentration of UO_2_^2+^. Inset: The linear calibration plot for UO_2_^2+^ detection. Error bars represent S.D. *n* = 3 independent experiments.

To explore the sensitivity, the fluorescence spectra of TFPT-BTAN-AO were measured at different UO_2_^2+^ concentrations (Fig. 4a). The fluorescence intensity of TFPT-BTAN-AO decreased with the increasing concentration of UO_2_^2+^ and 87% of the fluorescence was quenched with 20 μM UO_2_^2+^. In addition, the fluorescence response to UO_2_^2+^ was clearly observed under a 365 nm UV lamp. Figure 4b shows a good calibration curve for the fluorescence intensity of TFPT-BTAN-AO at 460 nm versus UO_2_^2+^ concentration (0.02–6.0 μM) with a high correlation coefficient of 0.993. The limit of detection of the TFPF-BTAN-AO was determined as 6.7 nM UO_2_^2+^ (Supplementary Fig. 13), well below the World Health Organization contamination limit for UO_2_^2+^ in drinking water (63 nM)^7^. Therefore TFPF-BTAN-AO can be used for high sensitivity detection of UO_2_^2+^.

### Interaction between TFPT-BTAN-AO and UO_2_^2+^

FT-IR and X-ray photoelectron spectroscopy (XPS) were applied to investigate the effective uranium-binding and fluorescence quenching. The FT-IR spectrum of TFPT-BTAN-AO after treatment with UO_2_^2+^ shows a new peak at 916 cm^−1^, which can be attributed to the vibration of O═U═O (Supplementary Fig. 14)^42^. In addition, the appearance of N-H bending vibration at 1613 cm^−1^ after treatment with UO_2_^2+^ indicates the presence of a chemisorption process^8^. After treating the TFPT-BTAN-AO with UO_2_^2+^, distinctive U 4 *f*-binding energy peaks emerged, revealing that UO_2_^2+^ was successfully loaded onto the TFPT-BTAN-AO (Supplementary Fig. 15)^43,44^.

**Fig. 5:**
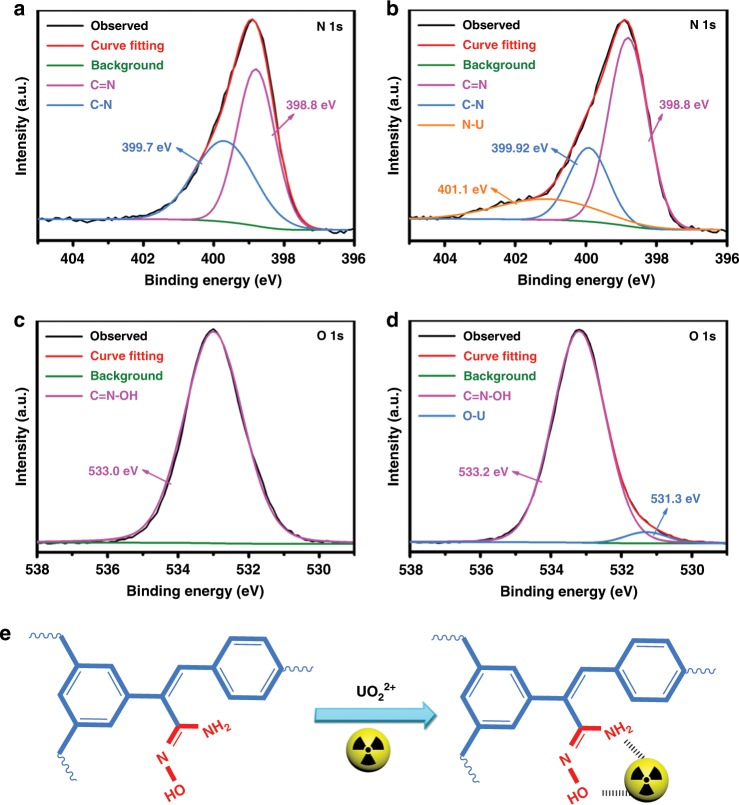
XPS data, fits, and interaction between TFPT-BTAN-AO and UO_2_^2+^. XPS spectra of the N 1 s region of TFPT-BTAN-AO **a** before and **b** after treatment with UO_2_^2+^. XPS spectra of the O 1 s region of TFPT-BTAN-AO **c** before and **d** after treatment with UO_2_^2+^. **e** Schematic diagram of the interaction between TFPT-BTAN-AO and UO_2_^2+^.

In the high-resolution XPS spectrum of N 1 s of the TFPT-BTAN-AO (Fig. 5a), the two binding energy peaks at 399.7 and 398.8 eV are assigned to C-N and C═N, respectively, which correspond to the nitrogen atoms in the TFPT-BTAN-AO framework^42,45^. After treatment with UO_2_^2+^, the N 1 s XPS spectrum of the TFPT-BTAN-AO was observed under the same measurement conditions (Fig. 5b). Comparing the N 1 s binding energy peaks in the two samples, a new N-U peak (401.1 eV) formed, and the peak located at 399.7 eV of the TFPT-BTAN-AO moved 0.22 eV to a higher binding energy after treatment with UO_2_^2+^. However, the N 1 s peaks at 398.8 eV in Fig. 5a, b show no shift after treatment with UO_2_^2+^, revealing that the nitrogen atoms of the C═N did not bind to UO_2_^2+^. Comparing the O 1 s peaks in the two samples (Fig. 5c, d), it is clearly observed that a new O-U peak (531.3 eV) formed, and the O 1 s core peak of the TFPT-BTAN-AO moved 0.2 eV to a higher binding energy after treatment with UO_2_^2+^. Based on the above results, it is speculated that the adsorption of UO_2_^2+^ onto the TFPT-BTAN-AO is a chemical process, and both the amino and hydroxyl groups in TFPT-BTAN-AO are coordinated with UO_2_^2+^ (Fig. 5e), similar to the previously reported binding mode^46^.

The quenching effect of UO_2_^2+^ on TFPT-BTAN-AO was further studied by time-resolved fluorescence spectroscopy. The fluorescence decay profile shows that TFPT-BTAN-AO has a lifetime of 3.1 ns (Supplementary Fig. 16, red curve). Upon addition of UO_2_^2+^, the lifetime decreased to 1.8 ns (blue curve), which is consistent with a decrease in fluorescence intensity. UO_2_^2+^ induced quenching of TFPT-BTAN-AO likely to proceed by a photoinduced electron transfer (PET) process from the framework to UO_2_^2+^.

### Efficient extraction of UO_2_^2+^

Given the very stable (-C═C-) bonds and abundant selective UO_2_^2+^-binding groups on the 1D channels, this material may serve for high performance UO_2_^2+^ extraction under harsh conditions. To test the importance of the regular porous structure for adsorbing UO_2_^2+^, an amorphous analog named POP-TB was synthesized and subjected to the same amidoximation to give POP-TB-AO (Supplementary Figs. 17–19). Both the COF-based materials and their amorphous POP analogs were demonstrated very similar FT-IR spectra and nitrogen element contents before and after the amidoximation process (Supplementary Figs. 20 and 21), indicating similar amidoxime functionalization processes.

**Fig. 6:**
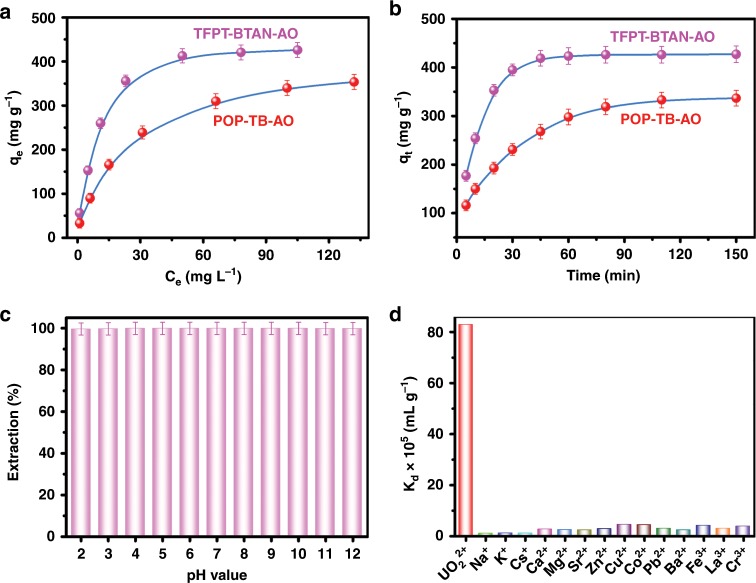
UO_2_^2+^ adsorption isotherms and kinetics investigations. **a** Adsorption isotherm of UO_2_^2+^ on TFPT-BTAN-AO and POP-TB-AO (pH 4.0). **b** Adsorption kinetics of UO_2_^2+^ on TFPT-BTAN-AO and POP-TB-AO (pH 4.0). **c** The removal efficiency of UO_2_^2+^ under different pH conditions. **d** The selective adsorption of the test ions. Error bars represent S.D. *n* = 3 independent experiments.

The adsorption isotherm of TFPT-BTAN-AO has a much steeper adsorption curve for UO_2_^2+^ (Fig. 6a), indicating that its affinity for UO_2_^2+^ is higher than that of corresponding POP-TB-AO^24^. All the adsorption experimental data are in good agreement with the Langmuir isotherm model, and the correlation coefficients are higher than 0.99 (Supplementary Fig. 22). Surprisingly, the maximum adsorption capacity for UO_2_^2+^ on TFPT-BTAN-AO (427 mg g^−1^) is much higher than that of POP-TB-AO (353 mg g^−1^), and is located among the top of different types of adsorbents (Supplementary Table 5). Importantly, the superior performance of TFPT-BTAN-AO exceeded all previously reported COFs for UO_2_^2+^ extraction, like COF-TpDb-AO (408 mg g^−1^)^1^, ACOF (169 mg g^−1^)^43^, and *o*-GS-COF (144.2 mg g^−1^)^3^. The uranium content loaded on the framework was calculated based on the ICP-MS results, this capacity means that 66.8% accessibility of the amidoxime groups in TFPT-BTAN-AO were used to extract UO_2_^2+^. The TFPT-BTAN-AO showed exceptional performance in extraction of UO_2_^2+^ in terms of saturated adsorption capacity as compared to POP-TB-AO, suggesting the important role of the adsorbent’s architecture. This surprisingly high saturation UO_2_^2+^ extraction capacity can be attributed to synergistic effect of the rich and even distribution of amidoxime groups on the pore walls and the higher accessibility of UO_2_^2+^ in the open 1D channel.

In addition to the high adsorption capacity, TFPT-BTAN-AO also extracted UO_2_^2+^ from aqueous solution more rapidly compared with POP-TB-AO (Fig. 6b). All the adsorption kinetics data are in good agreement with the pseudo-second-order model, and the correlation coefficients are higher than 0.995 (Supplementary Fig. 23). It is worth noting that TFPT-BTAN-AO can achieve a saturation capacity of about 98% within 45 min. This is in sharp contrast to the long contact times required for other uranium sorbent materials, which typically range from hours to days^7,47–49^. For comparison, POP-TB-AO took 85 min to reach 95% of its equilibrium adsorption capacity.

Apart from rapid equilibration, TFPT-BTAN-AO also has higher extraction capacity with an equilibrium capacity of 417 mg g^−1^, whereas POP-TB-AO only reaches 336 mg g^−1^ (Supplementary Fig. 24). Considering that TFPT-BTAN-AO and POP-TB-AO have similar chemical compositions, the high absorption capacity and rapid adsorption kinetics should be attributed to their different pore structures. The rapid kinetics of TFPT-BTAN-AO can be attributed to the hierarchical pores structure and the evenly and densely distributed chelating sites on the pore walls to facilitate rapid diffusion of UO_2_^2+^ throughout the framework (Supplementary Fig. 25). In contrast, the pore structure in POP-TB-AO is irregular, making it more susceptible to clogging, thus greatly impairing their adsorption performance.

For practical applications, extraction of UO_2_^2+^ under various harsh conditions is highly desirable. Considering that UO_2_^2+^ is mainly present in the acidic environment and hydrolysis occurs at a higher pH (Supplementary Fig. 26), adsorption experiments were performed at pH < 5.0. Supplementary Fig. 27 shows the adsorption capacity of UO_2_^2+^ on TFPT-BTAN-AO at different pH values. Obviously, the adsorption capacity increases as the system pH value rises from 1.0 to 4.0.

As nuclear fuel reprocessing and wastewater are usually treated under highly acidic conditions, it is necessary to evaluate the effect of solution acidity on UO_2_^2+^ extraction by TFPT-BTAN-AO (Supplementary Fig. 27). Surprisingly, TFPT-BTAN-AO showed high adsorption capacity in highly acidic media, the saturated capacity in 3.0 M nitric acid was calculated to be 128 mg g^−1^, this capacity is much higher than COF-IHEP1 (70 mg g^−1^, 2 M nitric acid)^50^. To further confirm the chemical stability and excellent uranium extraction performance of TFPT-BTAN-AO, the adsorption capacities of TFPT-BTAN-AO after treatment with water (100 °C), HCl (1 M), HNO_3_ (0.1–5.0 M), NaOH (1 M), and γ-ray irradiation (50 kGy, 200 kGy) were also studied (Supplementary Fig. 28). The results verified that the uranium extraction performance of TFPT-BTAN-AO was almost unchanged after treatment under various extreme conditions, indicating that TFPT-BTAN-AO has excellent stability and practical application potential. This feature is a significant advantage over imine-based COFs sorbents that typically suffer from the decomposition of structures under extreme conditions^1,3^.

In addition, the uranium concentration reduced from 9.952 ppm to 8.45 ppb and 6.17 ppb at pH values of 2 and 12, respectively (Fig. 6c). It is well below the US Environmental Protection Agency (EPA) uranium-containing wastewater discharge standard (30 ppb)^1^. To evaluate the affinity of TFPT-BTAN-AO toward UO_2_^2+^, additional selective extraction experiments with 9.952 ppm UO_2_^2+^ (*V*/*m* = 5000 mL g^−1^) were carried out. The calculated distribution coefficient *K*_*d*_ (8.3 × 10^6^ mL g^−1^) is much larger than the distinguishing standard (1.0 × 10^4^ mL g^−1^), which is generally considered to be a good adsorbent (Fig. 6d), indicating the excellent affinity of TFPT-BTAN-AO for UO_2_^2+^^47^. We attribute the enormous distribution coefficient to the specific affinity for UO_2_^2+^ provided by the 1D open channel amidoxime groups in TFPT-BTAN-AO. The above results indicate the superiority of TFPT-BTAN-AO as a promising candidate for uranium extraction.

### Reversible binding for regeneration

**Fig. 7:**
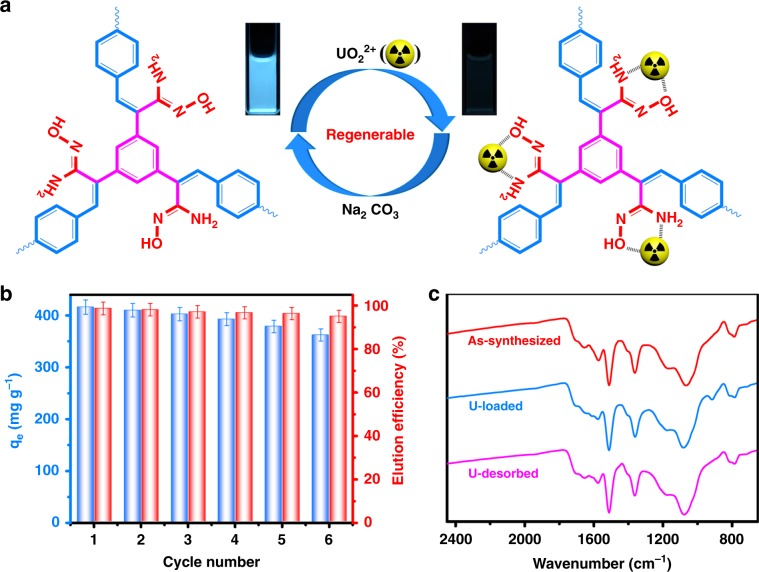
UO_2_^2+^ adsorption and regeneration investigations. **a** Schematic diagram of TFPT-BTAN-AO regeneration detection and extraction of UO_2_^2+^. **b** The UO_2_^2+^ adsorption capacity (left axis) and elution efficiency (right axis) of TFPT-BTAN-AO in six successive adsorption–desorption cycles. **c** FT-IR spectra of TFPT-BTAN-AO, TFPT-BTAN-AO after extraction of UO_2_^2+^, and TFPT-BTAN-AO after desorption of UO_2_^2+^ by the Na_2_CO_3_ (1.0 mol L^−1^) aqueous solution. Error bars represent S.D. *n* = 3 independent experiments.

One of the unique advantages of TFPT-BTAN-AO is that the very stable framework provides the most critical foundation for the reversible binding of UO_2_^2+^. We found that the fluorescence of TFPT-BTAN-AO can be easily recovered by Na_2_CO_3_ (1 M) aqueous solution (Fig. 7a). To confirm the practical reusability of TFPT-BTAN-AO as an adsorbent, we conducted multiple adsorption–desorption experiments and found that sodium carbonate has a high elution efficiency (>95%) even after six cycles (Fig. 7b). In addition, elution of UO_2_^2+^ with sodium carbonate did not affect the adsorption performance of TFPT-BTAN-AO, and maintained a high adsorption capacity (>87%) even after six cycles. More importantly, owing to the excellent stability of the framework, TFPT-BTAN-AO crystal structure and functional groups were well preserved after recycling, as evidenced by PXRD and FT-IR results (Supplementary Fig. 29 and Fig. 7c). It is worth noting that TFPT-BTAN-AO can be cycled at least six times without noticeable influence of response to UO_2_^2+^ or sensitivity, and this exceptional regeneration can be observed by the naked eye under a portable UV lamp (Supplementary Fig. 30). So far, this is the first demonstration of COF-based regenerable detection and extraction of UO_2_^2+^. The above results indicate that TFPT-BTAN-AO has great potential for simultaneous detection and extraction of UO_2_^2+^. Importantly, such regeneration is almost impossible for previously reported imine-based COFs adsorbents.

## Discussion

In summary, we have successfully developed a strongly fluorescent COF for real-time detection and efficient extraction of UO_2_^2+^. Different from previous COF sorbents relying on imine bond (-C═N-), our COF used very stable carbon–carbon double bonds (-C═C-). Our COF combines strong fluorescence, excellent stability, dense, and evenly distributed amidoxime groups, and highly accessible binding sites through the open 1D channel. With these advantages, TFPT-BTAN-AO achieved real-time sensitive detection, efficient extraction, and efficient regeneration by simply adding carbonate. Given the wealth of knowledge in designing contaminant-specific ligands, this strategy may extend to the detection and extraction of other environmental contaminants.

## Methods

### Materials

2,4,6-Tris(4-bromophenyl)-1,3,5-triazine, *n*-BuLi, hydroxylamine hydrochloride (NH_2_OH·HCl), DBU, and triethylamine were purchased from Saan Chemical Technology (Shanghai) Co., Ltd. Benzaldehyde and 1,3,5-tris(bromomethyl)benzene were purchased from Jilin Chinese Academy of Sciences-Yanshen Technology Co., Ltd. Mesitylene, 1,4-dioxane, acetone, chloroform (CHCl_3_), tetrahydrofuran (THF), N,N-dimethylformamide, sodium bicarbonate, sodium cyanide, *n*-hexane, *n*-BuOH, NaOH, EtONa, Cs_2_CO_3_, piperidine, pyridine, 1,2-dichlorobenzene, and MgSO_4_ were purchased from Sinopharm Chemical Reagent Co., Ltd. Ultrapure water was prepared from the Millipore system (18.25 MΩ-cm). All the purchased reagents were of analytical grade and used without further purification.

### Synthesis of model compound

To a 25 mL Pyrex tube, benzaldehyde (63.67 mg, 0.60 mmol), 2,2′,2″-(benzene-1,3,5-triyl)triacetonitrile (39.04 mg, 0.20 mmol), *o*-DCB (5 mL) and DBU aqueous solution (0.5 mL, 4 M) were added. The mixture was sonicated for 10 minutes, degassed by three freeze–pump–thaw cycles, sealed under vacuum and heated at 90 °C for 3 days. The reaction mixture was cooled to room temperature, the precipitate was filtered and washed several times with ethanol. The product was obtained as a pale-yellow solid. Yield: 72%. ^1^H NMR (CDCl_3_, *δ* (ppm)): 7.99 (m, 6 H, ArH), 7.96 (s, 3 H, Ar-H), 7.71 (s, 3 H, vinyl), 7.52 (m, 9 H, Ar-H). ^13^C NMR (DMSO-d^6^, *δ* (ppm)): 150.44, 140.99, 138.61, 136.29, 134.54, 134.24, 129.01, 122.75, 114.31. Elemental analysis: calculated C (86.25%), H (4.61%), N (9.14%) and observed C (86.09%), H (4.45%), N (9.03%).

### Synthesis of TFPT-BTAN COF

To a 25 mL Pyrex tube, 2,4,6-tris(4-formylphenyl)-1,3,5-triazine (78.68 mg, 0.20 mmol), 2,2′,2″-(benzene-1,3,5-triyl)triacetonitrile (39.04 mg, 0.20 mmol), *o*-DCB (5 mL) and DBU aqueous solution (0.5 mL, 4 M) were added. The mixture was sonicated for 10 minutes, degassed by three freeze–pump–thaw cycles, sealed under vacuum and heated at 90 °C for 5 days. The reaction mixture was cooled to room temperature, and a pale-yellow precipitate was collected by centrifugation, washed several times with methanol, CH_2_Cl_2_, and THF, respectively. It was then Soxhlet extracted in CH_2_Cl_2_ and THF for 24 h and dried under vacuum at 80 °C for 12 h to afford pale-yellow powder, 67% yield. Elemental analysis: calculated C (79.10%), H (5.53%), N (15.37%), and observed C (74.31%), H (5.72%), N (15.65%). For other conditions, follow the same experimental procedure to obtain TFPT-BTAN COF, as shown in the Supplementary Table 1.

### Synthesis of TFPT-BTAN-AO

The TFPT-BTAN (0.4 g) was swollen in absolute ethanol (40 mL) for 20 min, followed by the addition of NH_2_OH·HCl (1.0 g) and N(CH_2_CH_3_)_3_ (1.5 g). After stirring at 85 °C for 24 h, the mixture was filtered, washed with excess water and finally dried at 60 °C under vacuum. The pale-yellow solid obtained was expressed as TFPT-BTAN-AO. Elemental analysis: calculated C (66.96%), H (6.09%), N (19.52%), and observed C (65.31%), H (6.22%), N (19.75%).

### Synthesis of POP-TB

To a 25 mL Pyrex tube, 2,4,6-Tris(4-formylphenyl)-1,3,5-triazine (78.68 mg, 0.20 mmol), 2,2′,2″-(benzene-1,3,5-triyl)triacetonitrile (39.04 mg, 0.20 mmol), 1,4-dioxane (5 mL), and NaOH aqueous solution (0.5 mL, 4 M) were added. The mixture was sonicated for 10 minutes, degassed by three freeze–pump–thaw cycles, sealed under vacuum and heated at 90 °C for 5 days. The reaction mixture was cooled to room temperature, and a pale-yellow precipitate was collected by centrifugation, washed several times with methanol, CH_2_Cl_2_, and THF, respectively. It was then Soxhlet extracted in CH_2_Cl_2_ and THF for 24 h and dried under vacuum at 80 °C for 12 h to afford pale-yellow powder, 70% yield. Elemental analysis: observed C (74.16%), H (5.64%), N (15.53%).

### Synthesis of POP-TB-AO

The POP-TB (0.4 g) was swollen in absolute ethanol (40 mL) for 20 min, followed by the addition of NH_2_OH·HCl (1.0 g) and N(CH_2_CH_3_)_3_ (1.5 g). After stirring at 85 °C for 24 h, the mixture was filtered, washed with excess water and finally dried at 60 °C under vacuum. The pale-yellow solid obtained was expressed as POP-TB-AO. Elemental analysis: observed C (65.15%), H (6.13%), N (19.58%).

## Supplementary information

Supplementary Information

## Data Availability

All data are either provided in the Article and its Supplementary Information or are available from the corresponding author upon request.
