# Supplementary material for: Regenerable and stable sp2 carbon-conjugated covalent organic frameworks for selective detection and extraction of uranium
Source: Nat Commun. 2020 Jan 23;11:436. doi: 10.1038/s41467-020-14289-x (PMC6978342; doi:10.1038/s41467-020-14289-x)
Supplement: Supplementary file 1 — Supplementary Information [file 41467_2020_14289_MOESM1_ESM.pdf]

Supplementary Information

Regenerable and stable *sp*^{2} carbon-conjugated covalent organic frameworks for selective detection and extraction of uranium

Wei-Rong Cui,[1] Cheng-Rong Zhang,[1] Wei Jiang,[1] Fang-Fang Li,[1] Ru-Ping Liang,[1] Juewen Liu,[2] and Jian-Ding Qiu[1]*

[1]College of Chemistry, Nanchang University, Nanchang 330031, China

[2]Department of Chemistry, Waterloo Institute for Nanotechnology, University of Waterloo, Waterloo, Ontario, N2L 3G1, Canada

*Corresponding authors. Tel/Fax: +86-791-83969518. E-mail: jdqiu@ncu.edu.cn.

Supplementary Methods.

1. Characterisation methods

FT-IR spectra were recorded with a Bruker TENSOR 27 instrument. Powder X-ray diffraction (PXRD) data of the nanomaterials were collected on a Bruker AXS D8 Advance A25 Powder X-ray diffractometer (40 kV, 40 mA) using Cu Kα (λ=1.5406 Å) radiation. The morphology and size of the nanomaterials were imaged by a transmission electron microscopy (TEM, JEM-2010, JEOL). The fluorescence (FL) spectra were recorded on a FL spectrophotometer (F-7000, Hitachi). X-ray photoelectron spectroscopy (XPS) spectra were performed on a Thermo VG Multilab 2000X with Al Kα irradiation. The radiation stability of TFPT-BTAN-AO was investigated in a GAMMATOR M-38-2 (USA) irradiator with a ^{60}Co source (γ-ray). ^{1}H NMR and ^{13}C NMR spectra were checked by Varian instruments (400 MHz). Solid-state NMR experiments were performed on a Bruker WB Advance II 600 MHz NMR spectrometer. The ^{13}C CP/MAS NMR spectra were recorded with a 4-mm double-resonance MAS probe and with a sample spinning rate of 10.0 kHz; a contact time of 2 ms (ramp 100) and a pulse delay of 3 s were applied. The nitrogen adsorption and desorption isotherms were measured at 77 K using a Micromeritics ASAP 2020M system. The samples were outgassed at 120 °C for 8 h before the measurements. Surface areas were calculated from the adsorption data using Brunauer-Emmett-Teller (BET) methods. The pore-size-distribution curves were obtained via the non-local density functional theory (NLDFT) method. The thermal properties of the nanomaterials were evaluated using a STA PT1600 Linseis thermogravimetric analysis (TGA) instrument over the temperature range of 30 to 800 °C under nitrogen atmosphere with a heating rate of 10 °C/min. Metal ions concentrations were determined using an iCAP Q inductively coupled plasma mass spectrometry (ICP-MS, Thermo Fisher Scientific, USA).

2. Sensing experiments

Stock solutions. The ions stock solutions (1 mM) were prepared by dissolving the corresponding nitrate salts or sodium salts of Ca^{2+}, Cd^{2+}, Co^{2+}, Cr^{3+}, Pb^{2+}, Mn^{2+}, Ba^{2+}, Sr^{2+}, Cs^{+}, Al^{3+}, Ni^{2+}, Zn^{2+}, Mg^{2+}, Cu^{2+}, Fe^{3+}, Ce^{3+}, La^{3+}, Sm^{3+}, Gd^{3+}, Nd^{3+} and UO_2^{2+} in ultrapure water; and the stock solutions (1.0 M) of CO_3^{2-} was prepared in ultrapure water using Na_2CO_3. Stock solution of TFPT-BTAN-AO (0.2 mg/mL) was prepared by dispersion of TFPT-BTAN-AO in ultra-pure water.

Fluorescence sensing experiments. The stock solution of TFPT-BTAN-AO (100 μL) was added to the solution which contains different amounts of UO_2^{2+} (200 μL), then diluted to 2 mL with ultra-pure water in a quartz cuvette. TFPT-BTAN-AO was readily dispersed in ultra-pure water and the obtained suspension was almost transparent. The fluorescence spectra were recorded immediately after an appropriate aliquot of the stock solution of ions was added. All measurements were excited at λ_{ex} = 277 nm and the corresponding emission wavelengths were tested from λ_{em} = 350 to 650 nm unless otherwise stated. After the addition of the ions stock solutions, the shape of the emission spectra did not change. For the recycle tests, the stock solution of CO_3^{2-} was further added to remove UO_2^{2+} from TFPT-BTAN-AO-U, and the recycled TFPT-BTAN-AO was reused in the next UO_2^{2+} detection and removal. The measured fluorescence intensities were used to assess the degree of the recovery. Each test was repeated at least for three times to get concordant values.

3. Sorption experiments

The aqueous solutions of UO_2^{2+} with different concentrations were obtained by diluting the stock ions solution with the proper amount of ultra-pure water unless otherwise indicated. The pH levels of the solutions were adjusted by HNO_3 or NaOH aqueous solution. The concentrations of UO_2^{2+} during all the experiments were detected by ICP-MS for extra low concentrations. All the adsorption experiments were performed at ambient conditions.

UO_2^{2+} sorption isotherms. Considering that uranium in practical application

exists mainly in the strong acidic environment and hydrolysis occurs in higher pH value, adsorption isotherm experiments were carried out at pH 4.0. The adsorbent (5.0 mg) was added to a 25 mL aqueous solution with different concentrations of UO_2^{2+} (pH = 4.0). The adsorbent was completely suspended by sonication, and then the mixture was vigorously stirred overnight, by which time it was assumed that adsorption equilibrium had been reached. The treated solution was filtered through a 0.22 μm membrane filter. The supernatant was analyzed using ICP-MS analysis to determine the remaining UO_2^{2+} concentration. The adsorbed amount at equilibrium (q_e, mg g^{-1}) was calculated by $q_e = (C_0 - C_e)/m \times V$, where V is the volume of the treated solution (L), m is the amount of used adsorbent (g), and C_0 and C_e are the initial concentration and the final equilibrium concentration of UO_2^{2+} (mg L^{-1}), respectively.

The experimental data was fitted using the Langmuir isotherm model. It can be depicted as follows: $q_e = q_m bC_e/(1 + bC_e)$, where b represent the Langmuir constant (L mg^{-1}), C_e represent the equilibrium concentration of metal ions (mg L^{-1}), q_m represent the monolayer adsorption capacity (mg g^{-1}), q_e represent the equilibrium adsorption capacity (mg g^{-1}).

UO_2^{2+} sorption kinetics. Considering that uranium in practical application exists mainly in the strong acidic environment and hydrolysis occurs in higher pH value, adsorption kinetics experiments were carried out at pH 4.0. The adsorbent (5.0 mg) was added to an Erlenmeyer flask containing UO_2^{2+} solution (200 mL) at 19.9 ppm and pH = 4.0. The adsorbent was completely suspended by sonication, and then the mixture was vigorously stirred for different times. The treated solution was filtered through a 0.22 μm membrane filter, and the filtrate was collected and analyzed by using ICP-MS to determine the remaining UO_2^{2+} content.

The experimental data was fitted using Pseudo-second-order kinetic model. It can be expressed as follows: $t/q_t = 1/K_2 q_e^2 + t/q_e$, where q_t and q_e represent the adsorbed amount (mg g^{-1}) at time and at equilibrium t (min), respectively, k_2 represent the Pseudo-second-order rate constant of adsorption (g mg^{-1} min^{-1}).

The distribution coefficient (K_d) value as used for the determination of the

affinity and selectivity of sorbents for UO_2^{2+} (mL g^{-1}), is given by the equation (1):

$$K_d = \frac{(C_0 - C_e)}{C_e} \times \frac{V}{m} \quad (1)$$

Where V is the volume of the treated solution (mL), m is the amount of used adsorbent (g), and C_0 and C_e are the initial concentration and the final equilibrium concentration of UO_2^{2+} (mg L^{-1}), respectively. In the present work, 9.952 ppm uranium aqueous solutions (pH=4.0) were treated by various adsorbents overnight at a V/m ratio of 5000 mL g^{-1}.

UO_2^{2+} adsorption capacity under different conditions. The adsorbent (5.0 mg) was added to an Erlenmeyer flask containing 19.9 ppm UO_2^{2+} solution (200 mL) under different conditions. The adsorbent was completely suspended by sonication, and then the mixture was vigorously stirred at room temperature for 3h. The treated solution was filtered through a 0.22 μm membrane filter, and the filtrate was collected and analyzed by using ICP-MS to determine the remaining UO_2^{2+} content. The adsorption capacity at equilibrium (q_e, mg g^{-1}) was calculated by $q_e = (C_0 - C_e)/m \times V$, where V is the volume of the treated solution (L), m is the amount of used adsorbent (g), and C_0 and C_e are the initial concentration and the final equilibrium concentration of UO_2^{2+} (mg L^{-1}), respectively.

UO_2^{2+} extraction at different pH values. The adsorbent (5.0 mg) was added to a glass vial containing UO_2^{2+} solution (25 mL) at 9.952 ppm and different pH values. The mixtures were stirred at room temperature for 3h, filtered through a 0.22 μm membrane filter, and the filtrate was collected and analyzed by using ICP-MS to determine the remaining UO_2^{2+} content. The percentage removal of UO_2^{2+} was calculated as follows: Removal % = $(C_0 - C_e)/C_0 \times 100\%$, C_0 and C_e are the initial concentration and the final equilibrium concentration of UO_2^{2+} (mg L^{-1}), respectively.

4. Stability and recyclability test

Stability test. TFPT-BTAN-AO was exposed to the γ-ray irradiation for two doses (50 kGy, 200 kGy), or immersed in water (100 °C), HCl (1 M), NaOH (1 M),

and HNO_3 (0.1 M, 0.5 M, 1.0 M, 3.0 M, and 5.0 M) for 12h, respectively. The mixture was then filtered and washed with ultra-pure water till the supernatant became neutral and dried under vacuum at 60 °C. Then, the FT-IR spectra and PXRD patterns were obtained.

Recyclability test. After one run of adsorption, TFPT-BTAN-AO was regenerated by treatment with 60 mL Na_2CO_3 (1 M) solution and shaken for 24 h. The resulting suspension was filtered and washed with ultra-pure water till the supernatant became neutral. After being dried under vacuum, the resultant material was used for another adsorption experiment. It was found that after six consecutive cycles TFPT-BTAN-AO still showed excellent uranium uptake.

5. Synthesis of monomers

Synthesis of 2,2',2''-(benzene-1,3,5-triyl)triacetonitrile (BTAN). Prepared as reported,[1] to a solution of 1,3,5-tris(bromomethyl)benzene (3.02 g, 8.50 mmol) in THF (25 mL) was added sodium bicarbonate (saturated solution, 30 mL) and sodium cyanide (4.17 g, 85.0 mmol) followed by 30 mL water. The solution was left to stir for 3 days after which it is was acidified with 1 M HCl. THF was removed via rotary evaporation to leave behind 1.50 g (90%) of an off-white solid. ^{1}H NMR ($CDCl_3$, δ (ppm)): 7.31 (s, 3H), 3.80 (s, 6H), IR: ν 2239 cm^{-1}(C≡N).

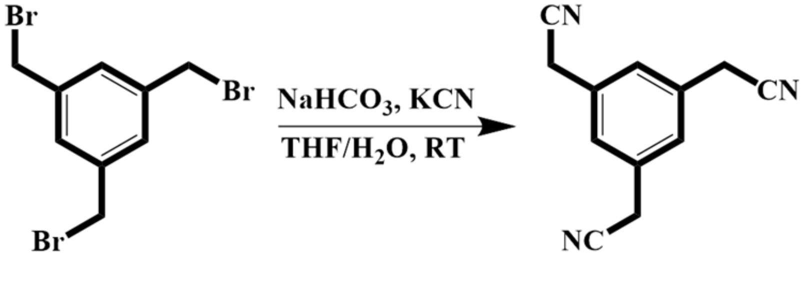

Synthesis of 2,4,6-Tris(4-formylphenyl)-1,3,5-triazine (TFPT). TFPT was synthesized via the method reported with slight modifications.[2] 2,4,6-Tris(4-bromophenyl)-1,3,5-triazine (1.48 g, 2.71 mmol) was dissolved in dry THF (200 mL) under a N_2 atmosphere. To the stirred solution, *n*-BuLi was added

dropwise (2.5 M in *n*-hexane, 11 mL, 27.5 mmol) at −78 °C. The temperature was allowed to rise to −60 °C and stirred for 3 h. The obtained green solution was treated with anhydrous *N,N*-dimethylformamide (DMF) (4.19 mL, 54.2 mmol) at −78 °C. The mixture was stirred overnight, while the temperature was allowed to rise to 25 °C. The milky opaque mixture was acidified with aqueous 3 M HCl (46 mL). The organic volatiles were partially removed by evaporation under reduced pressure, and the product was extracted with $CHCl_3$. The organic phase was washed with water, dried over $MgSO_4$, and filtered. Volatiles were removed under reduced pressure, and the crude light yellow product was further purified by silica gel column using $CHCl_3$ as eluent to afford colorless crystals (778 mg, 72%). ^1H NMR (DMSO-d^6, δ (ppm)): 10.19 (s, 3H, CHO), 8.96 (d, J = 8.4 Hz, 6H, ArH), 8.18 (d, J = 8.1, 6H, ArH).

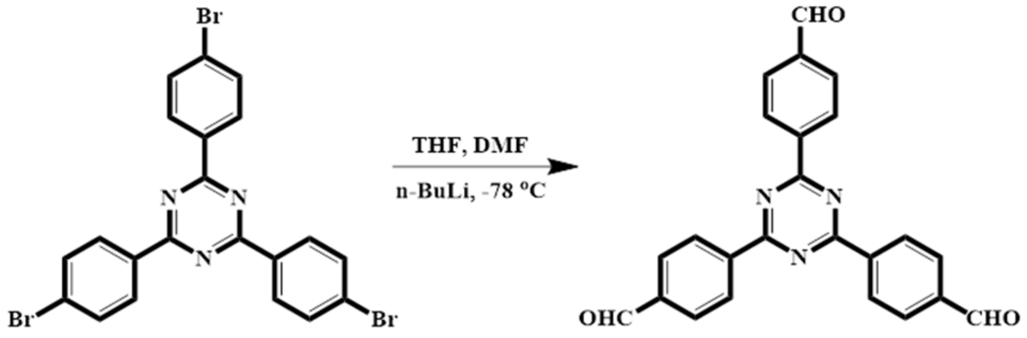

Supplementary Figures and Tables

Reaction condition optimization. In order to synthesize TFPT-BTAN with high crystallinity, we have optimized the reaction condition by changing the solvents, catalysts, concentrations of catalyst and temperatures. After many different trials, we successfully get the right condition to obtain high crystallinity TFPT-BTAN COF.

Supplementary Table 1 | Synthesis of the TFPT-BTAN under variable conditions.

Conditions	Ratio	Temperature	Catalyst	Product
Mesitylene/ 1,4-dioxane	1/1	90 °C	NaOH	Amorphous polymer
Mesitylene/ 1,4-dioxane	1/1	90 °C	Cs_2CO_3	Amorphous polymer
Mesitylene/ 1,4-dioxane	1/1	90 °C	EtONa	Amorphous polymer
Mesitylene/ 1,4-dioxane	1/1	90 °C	DBU	Poor crystallinity
Mesitylene/ 1,4-dioxane	1/1	90 °C	TEA	No product
Mesitylene/ 1,4-dioxane	1/1	90 °C	Piperidine	No product
Mesitylene/ 1,4-dioxane	1/1	90 °C	Pyridine	No product
o-DCB/ *n*-BuOH	1/1	90 °C	NaOH	Amorphous polymer
o-DCB/ *n*-BuOH	1/1	90 °C	Cs_2CO_3	Amorphous polymer
o-DCB/ *n*-BuOH	1/1	90 °C	EtONa	Amorphous polymer
o-DCB/ *n*-BuOH	1/1	90 °C	DBU	Poor crystallinity
o-DCB/ *n*-BuOH	1/1	90 °C	TEA	Poor crystallinity
o-DCB/ *n*-BuOH	1/1	90 °C	Piperidine	No product
o-DCB/ *n*-BuOH	1/1	90 °C	Pyridine	No product

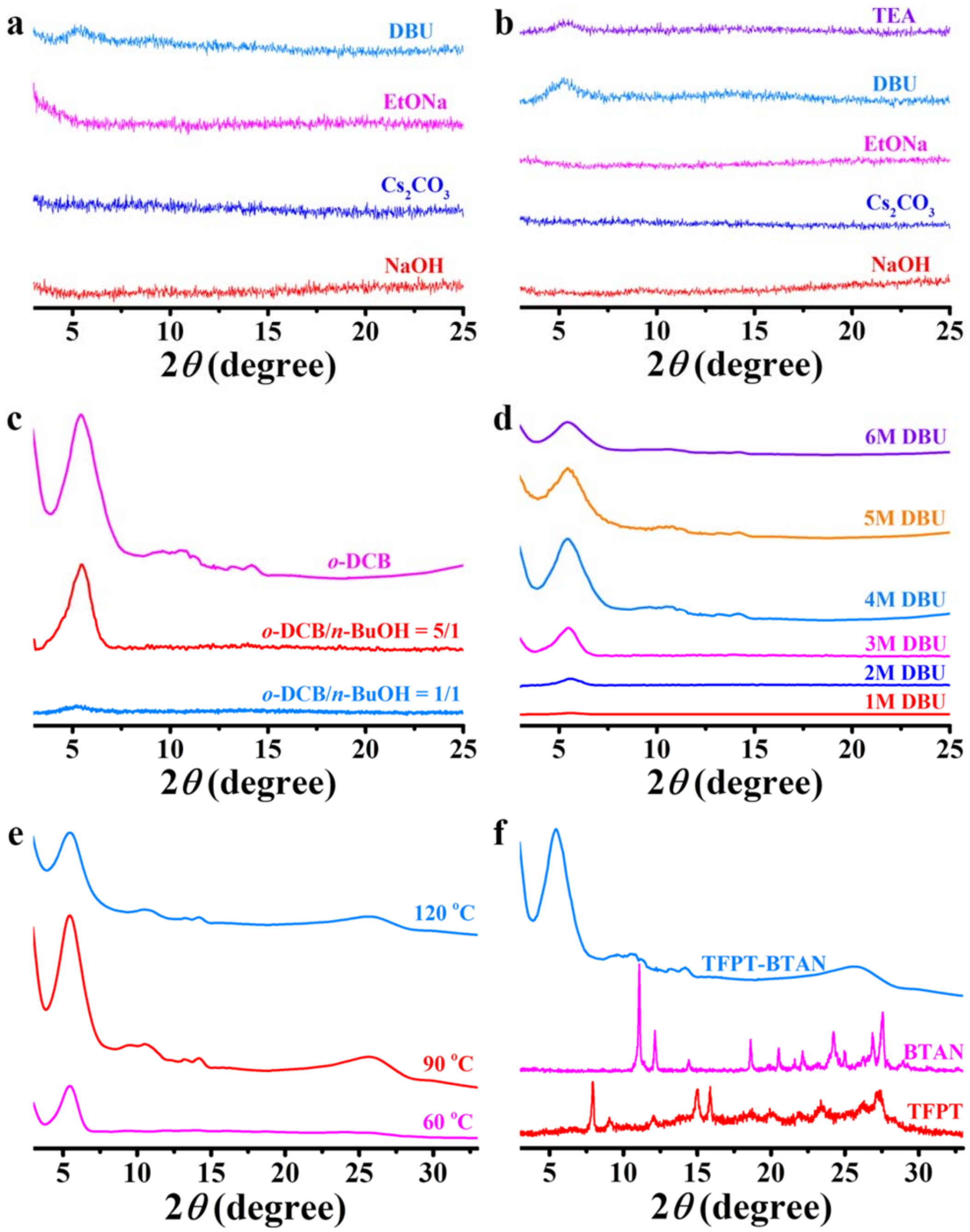

Supplementary Figure 1 | PXRD patterns of attempts to synthesize TFPT-BTAN under different reaction conditions (data collected on Bruker AXS D8 Advance A25 Powder X-ray diffractometer): (a) reaction conditions of different catalysts in the mixed solvents of Mesitylene/1,4-dioxane, (b) reaction conditions of different catalysts in the mixed solvents of *o*-DCB/*n*-BuOH, (c) reaction conditions with different solvent ratio, (d) reaction conditions with different concentration of catalyst, (e) reaction conditions with different temperature. (f) PXRD patterns of TFPT, BTAN, and TFPR-BTAN.

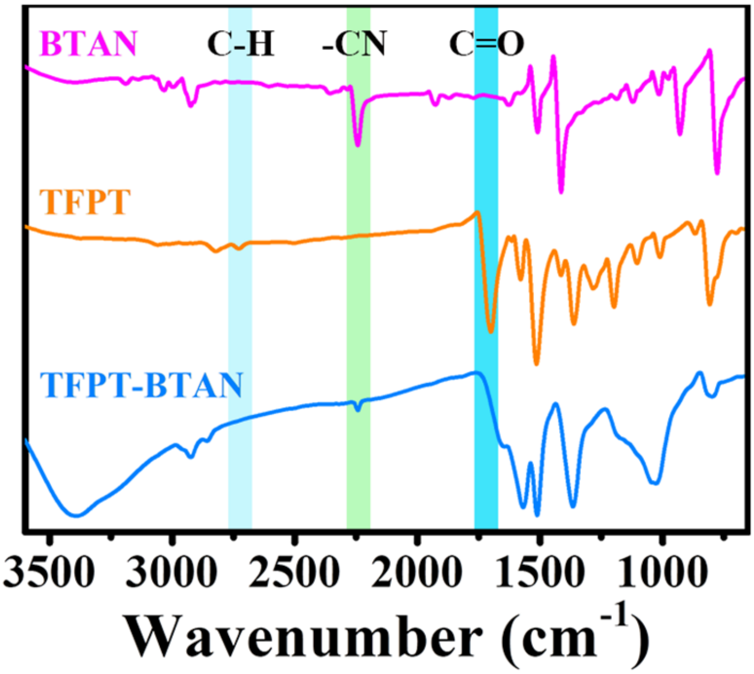

Supplementary Figure 2 | FT-IR spectra of BTAN, TFPT, and TFPT-BTAN.

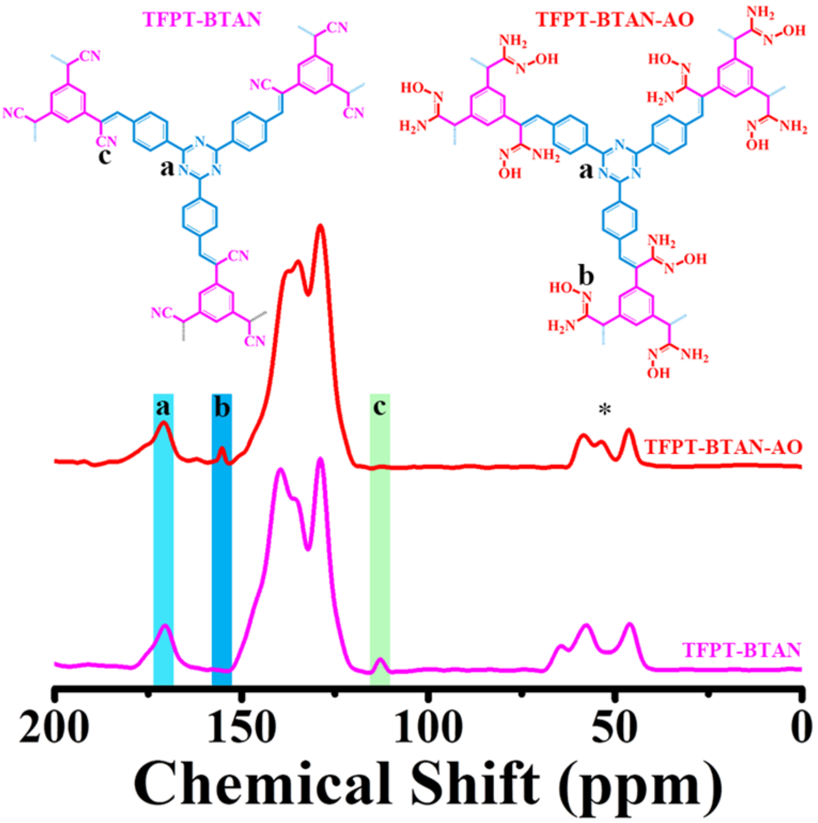

Supplementary Figure 3 | Solid-state ^{13}C CP/MAS NMR spectra of TFPT-BTAN and TFPT-BTAN-AO. Spinning side bands are marked with asterisk (*).

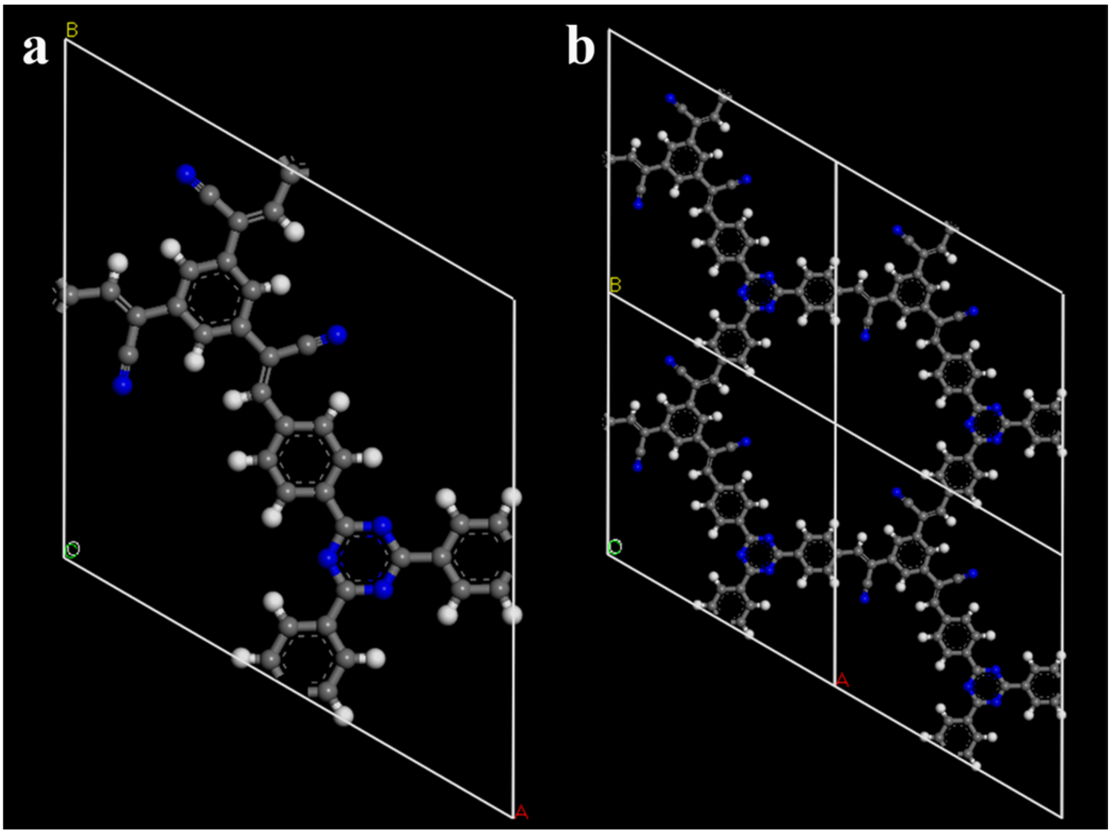

Supplementary Figure 4 | (a) Unit cell and (b) Eclipsed crystal lattice packing of TFPT-BTAN.

Supplementary Table 2 | Fractional atomic coordinates for the unit cell of TFPT-BTAN (Eclipsed).

**TFPT-BTAN**<br>**Hexagonal; P-6**<br>**a = b = 19.1509 Å, c = 3.4617 Å**<br>**α = β = 90°, γ = 120°**			
**Atom**	x (Å)	y (Å)	z (Å)
**N1**	0.70866	0.29392	0.5
**C2**	0.74867	0.37564	0.5
**C3**	0.58362	0.16182	0.5
**C4**	0.49367	0.3737	0.5
**C5**	0.4504	0.41462	0.5
**C6**	0.48976	0.49956	0.5
**C7**	0.57492	0.54198	0.5
**C8**	0.6182	0.50092	0.5
**C9**	0.43654	0.53661	0.5

**C10**	0.45543	0.61535	0.5
**C11**	0.39154	0.64063	0.5
**C12**	0.4153	0.72333	0.5
**C13**	0.53857	0.67945	0.5
**N14**	0.6046	0.73211	0.5
**H15**	0.4609	0.3084	0.5
**H16**	0.38516	0.37909	0.5
**H17**	0.61043	0.60632	0.5
**H18**	0.68349	0.53552	0.5
**H19**	0.37412	0.49115	0.5
**H20**	0.47847	0.76577	0.5
**N21**	0.70608	0.41473	0.5
**C22**	0.62436	0.37303	0.5
**C23**	0.83818	0.4218	0.5
**C24**	0.6263	0.11998	0.5
**C25**	0.58538	0.03577	0.5
**C26**	0.50044	0.99019	0.5
**C27**	0.45802	0.03293	0.5
**C28**	0.49908	0.11729	0.5
**C29**	0.46339	0.89992	0.5
**C30**	0.38465	0.84008	0.5
**C31**	0.35937	0.75091	0.5
**C32**	0.27667	0.69197	0.5
**C33**	0.32055	0.85912	0.5
**N34**	0.26789	0.87249	0.5
**H35**	0.6916	0.1525	0.5
**H36**	0.62091	0.00607	0.5
**H37**	0.39368	0.00411	0.5
**H38**	0.46448	0.14797	0.5
**H39**	0.50885	0.88296	0.5
**H40**	0.23423	0.71271	0.5
**N41**	0.58527	0.29134	0.5
**C42**	0.62697	0.25133	0.5
**C43**	0.5782	0.41638	0.5
**C44**	0.88002	0.50633	0.5
**C45**	0.96423	0.5496	0.5
**C46**	0.00981	0.51024	0.5
**C47**	0.96707	0.42508	0.5
**C48**	0.88271	0.3818	0.5
**C49**	0.10008	0.56346	0.5
**C50**	0.15992	0.54457	0.5

**C51**	0.24909	0.60846	0.5
**C52**	0.30803	0.5847	0.5
**C53**	0.14088	0.46143	0.5
**N54**	0.12751	0.3954	0.5
**H55**	0.8475	0.5391	0.5
**H56**	0.99393	0.61484	0.5
**H57**	0.99589	0.38957	0.5
**H58**	0.85203	0.31651	0.5
**H59**	0.11704	0.62588	0.5
**H60**	0.28729	0.52153	0.5

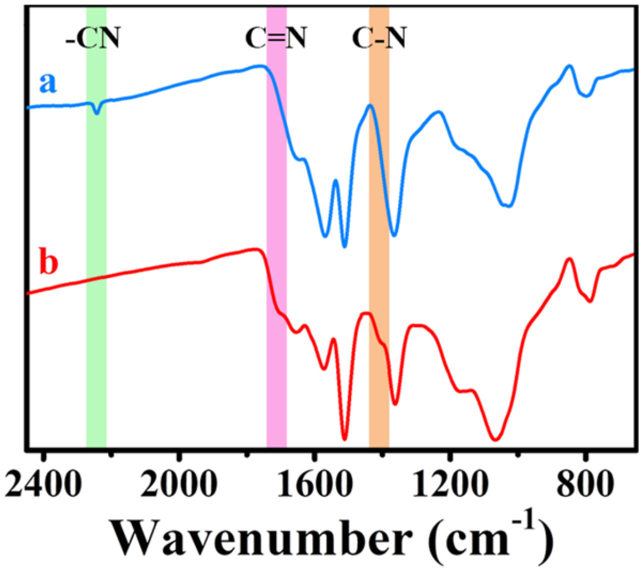

Supplementary Figure 5 | FT-IR spectra of TFPT-BTAN (a), TFPT-BTAN-AO (b).

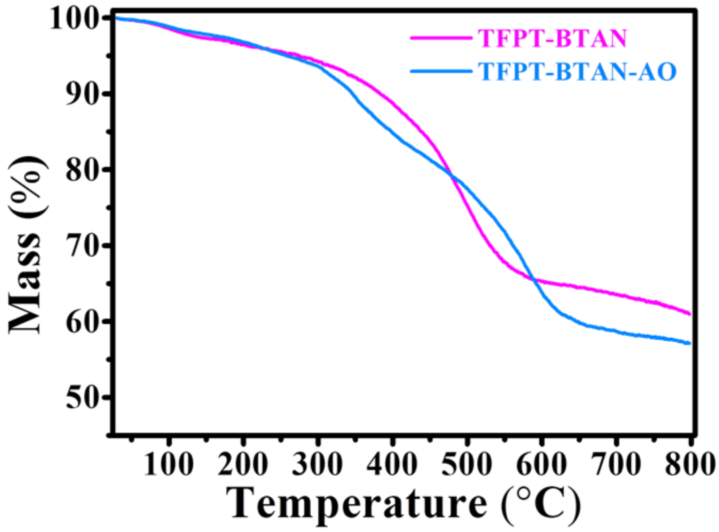

Supplementary Figure 6 | TGA curves of TFPT-BTAN and TFPT-BTAN-AO.

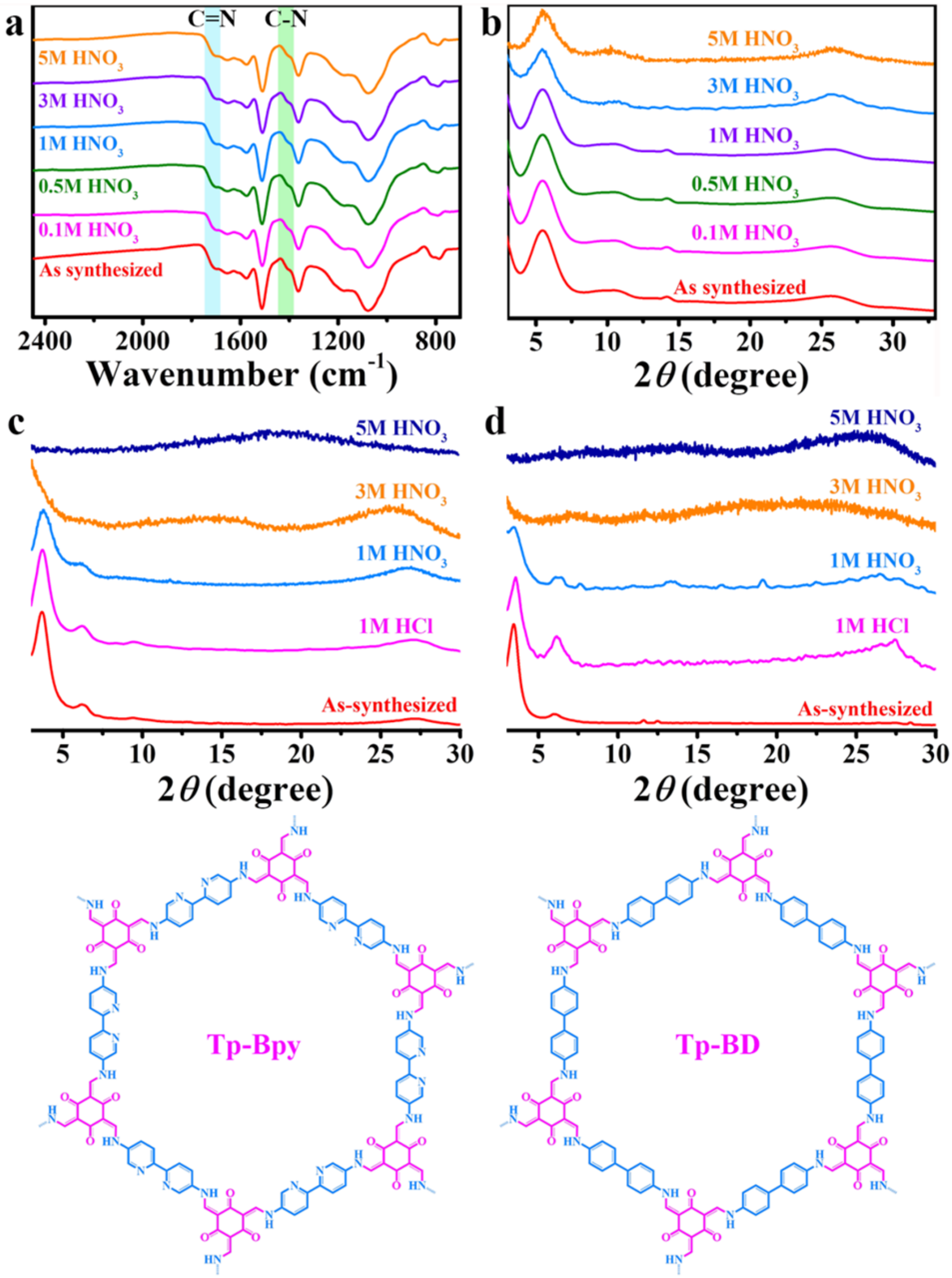

Supplementary Figure 7 | (a) The FT-IR spectra, and (b) PXRD profiles of TFPT-BTAN-AO before and after treatment with different concentrations of nitric acid. PXRD patterns of Tp-Bpy (c) and Tp-BD (d) before and after treatment under different conditions. Two β-ketoenamine COFs (Tp-Bpy and Tp-BD) were synthesized by the reported method.[16] The crystallinity of Tp-Bpy and Tp-BD was completely destroyed after treatment with high concentrations of nitric acid (3.0 M and 5.0 M), while our TFPT-BTAN-AO maintained good crystallinity and stability.

Supplementary Table 3 | The residual mass of the TFPT-BTAN-AO after treatment with different concentrations of nitric acid.

**Concentration (mol/L)**	**0.1**	**0.5**	**1.0**	**3.0**	**5.0**
**Before (mg)**	20.0	20.0	20.0	20.0	20.0
**After (mg)**	19.8	19.6	19.3	18.9	18.7

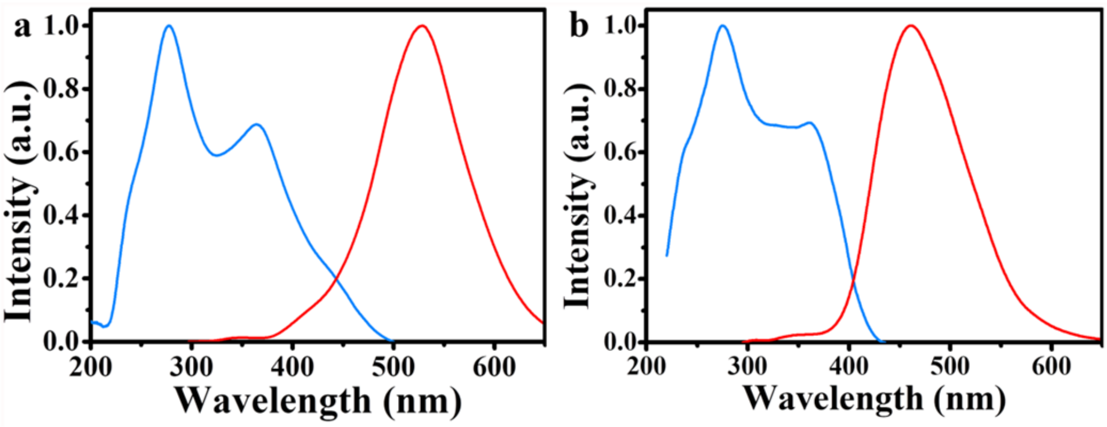

Supplementary Figure 8 | Normalized fluorescence excitation (blue) and emission (red) spectra of TFPT-BTAN (a) and TFPT-BTAN-AO (b) dispersed in water.

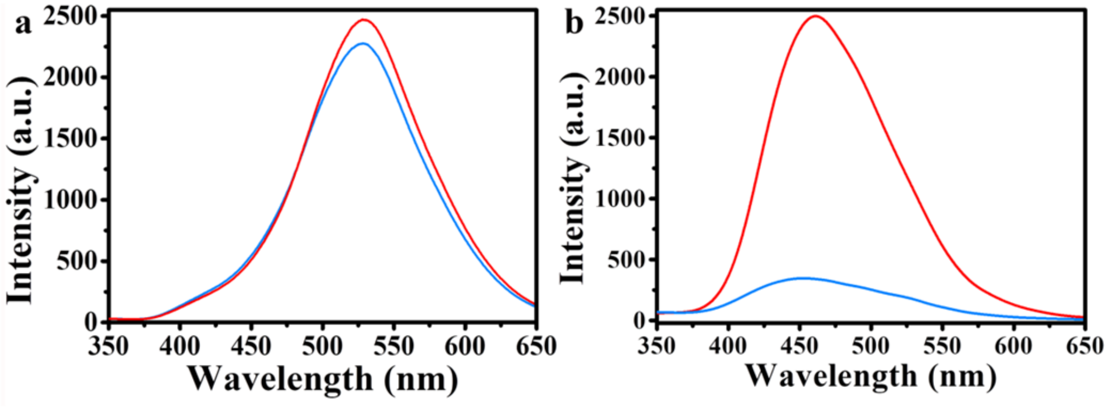

Supplementary Figure 9 | Fuorescence emission spectra of TFPT-BTAN (a) and TFPT-BTAN-AO (b) before and after addition of UO_2^{2+} (20 μM).

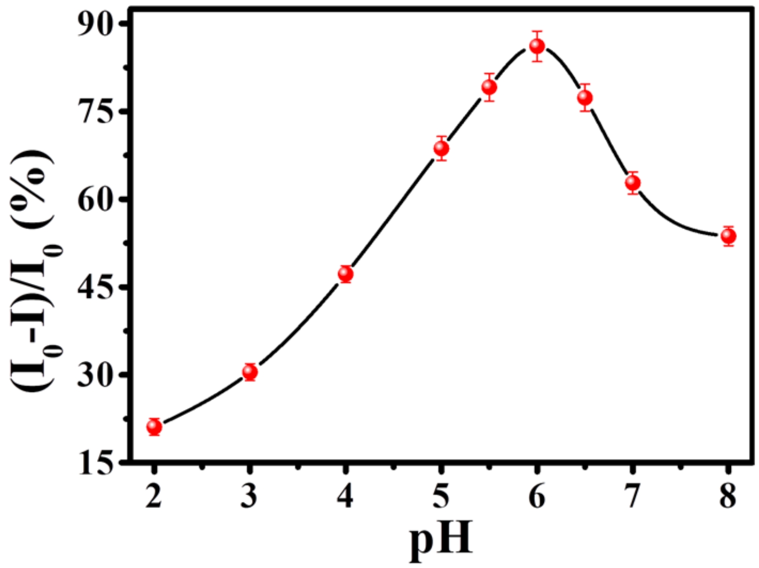

Supplementary Figure 10 | Effect of pH on the quenching ratio $[(I_0 - I)/I_0]\%$ of TFPT-BTAN-AO in the presence of 20 μM UO_2^{2+}. Error bars represent S.D. n =3 independent experiments.

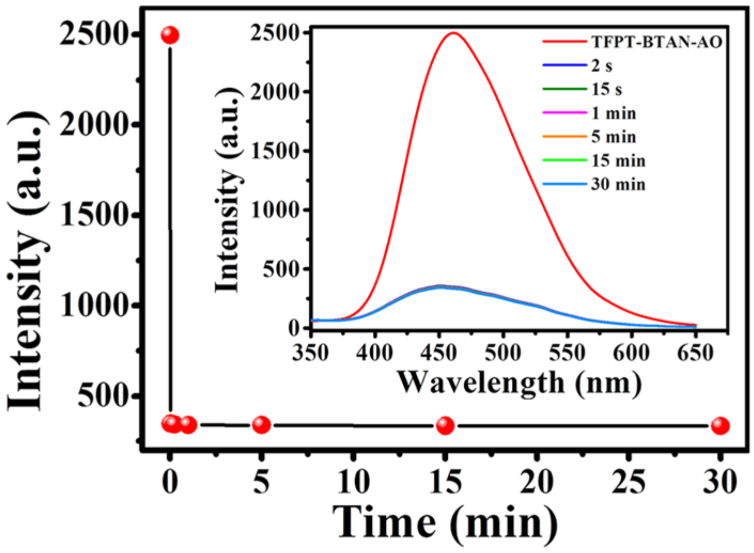

Supplementary Figure 11 | The time-dependent fluorescence intensity of TFPT-BTAN-AO upon addition of UO_2^{2+} (20 μM) was tested within 30 minutes.

Supplementary Table 4 | The response time comparison of representative detection methods for UO_2^{2+}.

System	Detection method	Response time	Ref.
Ag-rGO sheets	SERS	240 min	3
DNA-based voltammetric biosensor	Electrochemical	5-60 min	4
4,4′-Diaminophenylmethane	Absorption	30 min	5
CdS-MAA-TU	Fluorescence	10 min	6
Diglycolamide-capped quantum dots	Fluorescence	10 min	7
Tetraphenylethene-based sensor	Fluorescence	10 min	8
Metal-organic framework	Fluorescence	5 min	9
Dispersive liquid-liquid microextraction	ICP-MS	5 min	10
Amidoximated polymer	Fluorescence	200 s	11
Homogenous liquid-liquid microextraction	ICP-MS	3 min	12
N,P-CQDs	Fluorescence	2 min	13
CdTe-Quantum dots	Fluorescence	120 s	14
HOPO-modified CQDs	Fluorescence	30 s	15
TFPT-BTAN-AO	Fluorescence	2 s	This work

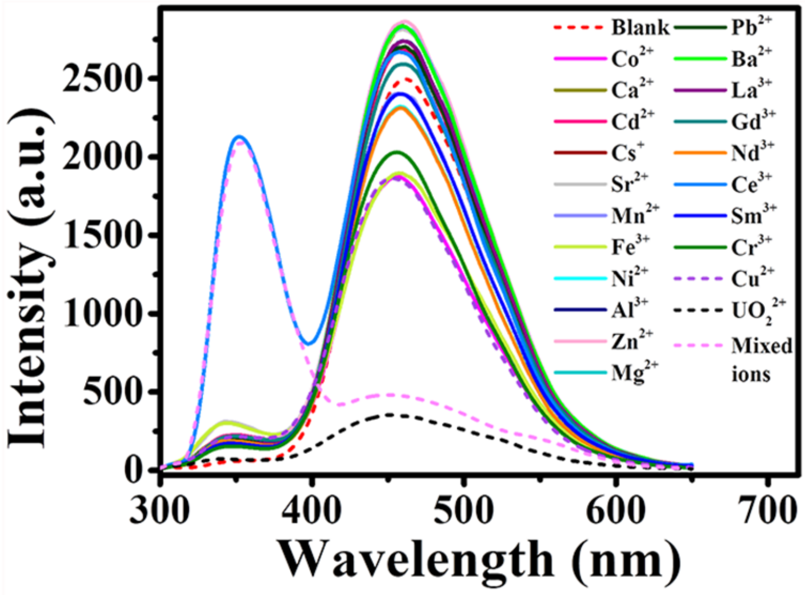

Supplementary Figure 12 | The emission spectra of TFPT-BTAN-AO in the presence of various cations and mixed ions. Concentrations of UO_2^{2+} and other metal ions were 20 μM and 50 μM, respectively.

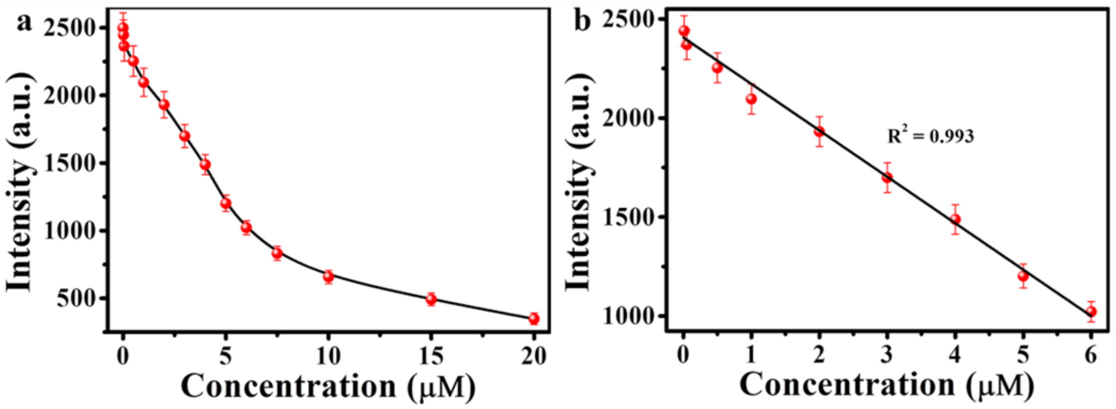

Supplementary Figure 13 | (a) PL intensity at 460 nm corresponding to various concentration of UO_2^{2+}. (b) The linear calibration plot of UO_2^{2+} for TFPT-BTAN-AO in water (λ_{ex} = 277 nm). The corresponding limit of detection (LOD) was determined as 6.7 nM using the equation LOD = 3 × S.D./k,[16] where k is the slope of the curve equation, and S.D. represents the standard deviation for the TFPT-BTAN-AO solution intensity in the absence of UO_2^{2+}. Error bars show the standard deviations of three experiments.

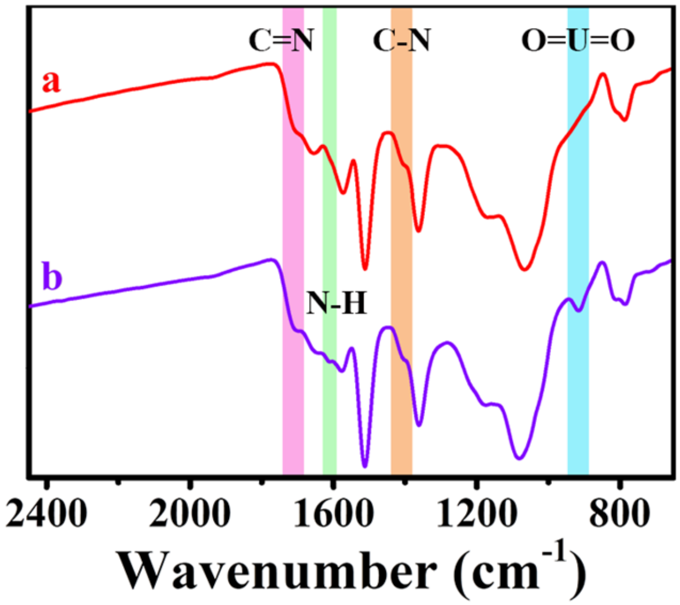

Supplementary Figure 14 | FT-IR spectra of TFPT-BTAN-AO (a), TFPT-BTAN-AO-U (b). The significant shift of the antisymmetric vibration of O=U=O in TFPT-BTAN-AO (916 cm^{-1}) in comparison with that in $UO_2(NO_3)_2\cdot6H_2O$ (~960 cm^{-1})[17] from the FT-IR studies further confirms that strong interactions exist between the UO_2^{2+} and TFPT-BTAN-AO.

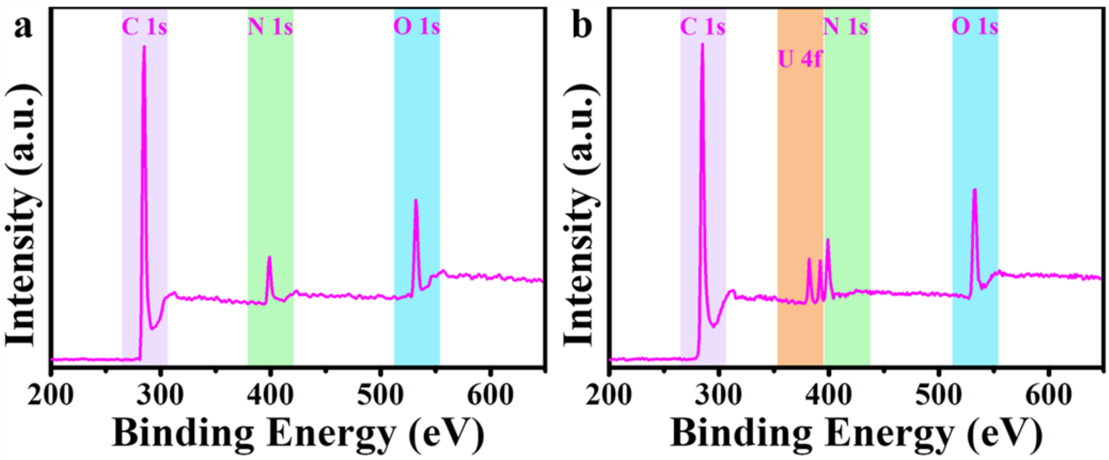

Supplementary Figure 15 | XPS survey spectra of TFPT-BTAN-AO before (a) and after treatment with UO_2^{2+} (b).

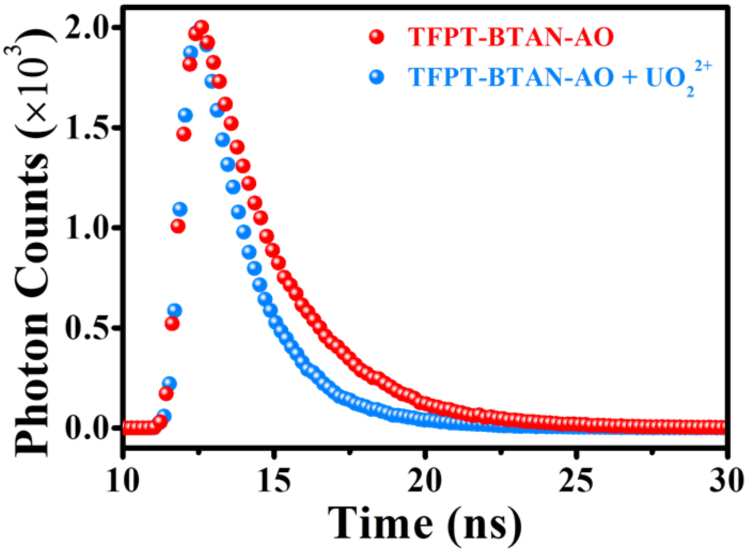

Supplementary Figure 16 | Fluorescence decay profiles (λ_{ex} = 277 nm and λ_{em} = 460 nm) of the TFPT-BTAN-AO itself and in the presence of UO_2^{2+} (20 μM) in water.

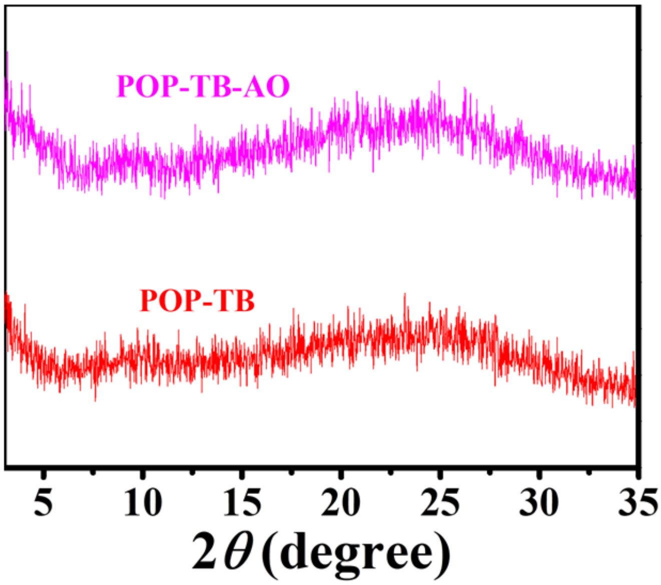

Supplementary Figure 17 | XRD patterns of POP-TB and POP-TB-AO.

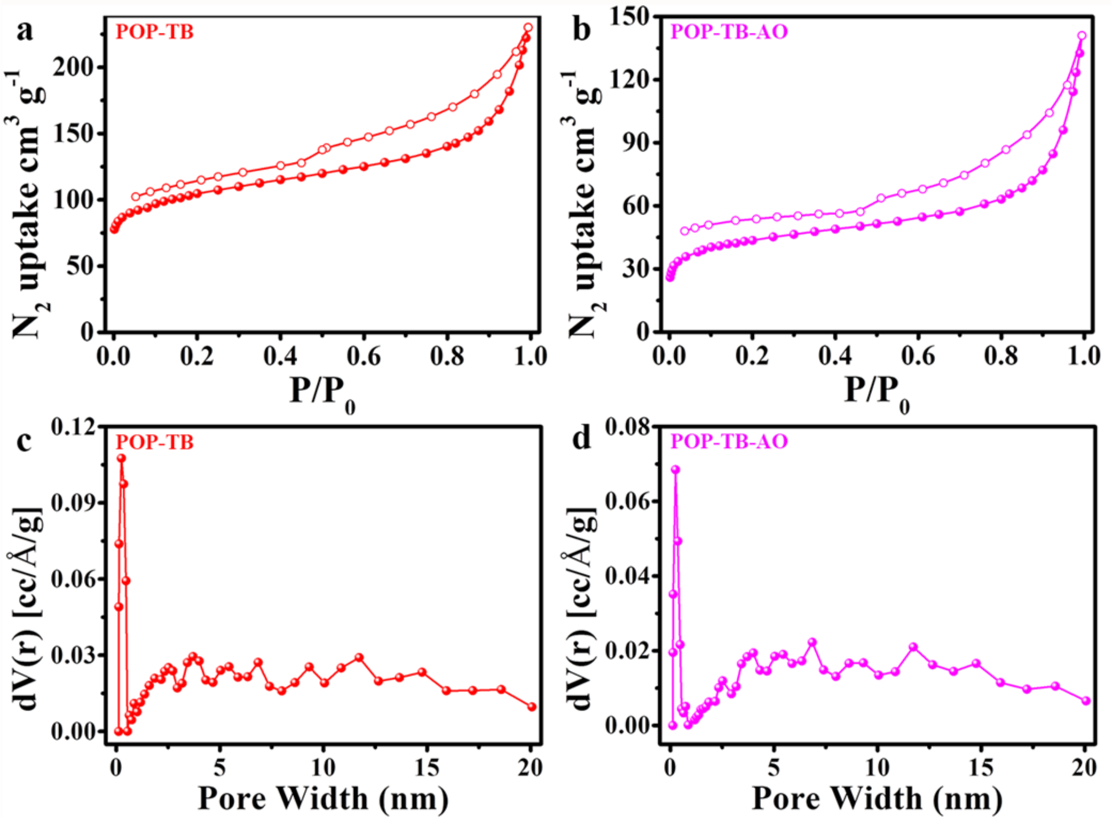

Supplementary Figure 18 | Nitrogen adsorption-desorption isotherms of POP-TB (a), and POP-TB-AO (b). The pore size distributions of POP-TB (c), and POP-TB-AO (d) calculated from non-local density functional theory. The BET surface areas of POP-TB and POP-TB-AO were calculated to be 276 and 142 m^2/g, respectively.

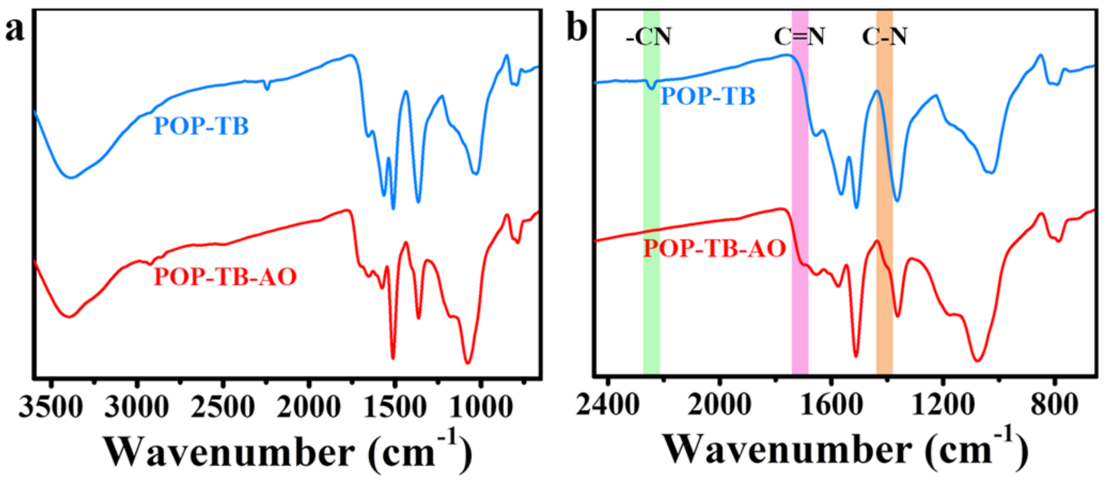

Supplementary Figure 19 | (a) FT-IR spectra of POP-TB and POP-TB-AO. (b) Zoomed FT-IR spectra of POP-TB and POP-TB-AO.

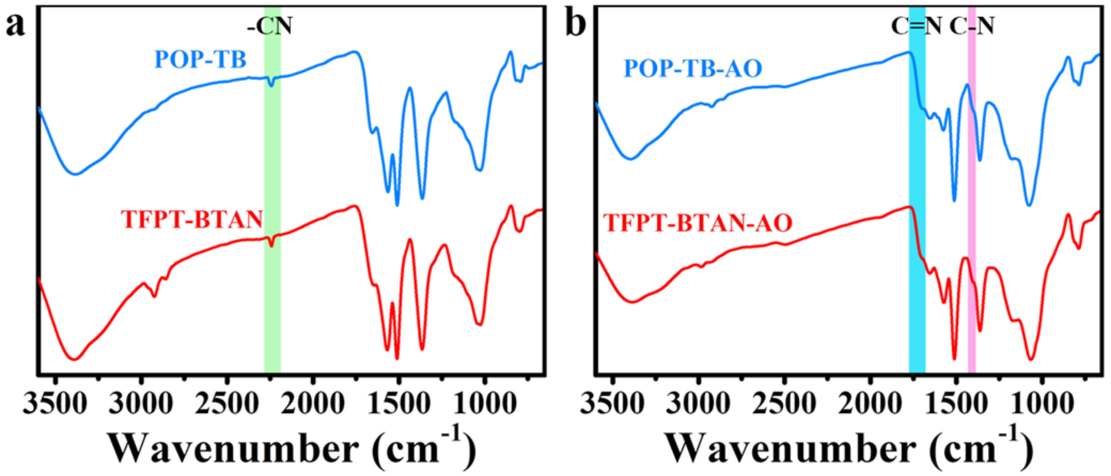

Supplementary Figure 20 | (a) FT-IR spectra of POP-TB and TFPT-BTAN. (b) FT-IR spectra of POP-TB-AO and TFPT-BTAN-AO. FT-IR spectra of POPs and COFs are very similar, indicating that their chemical composition is similar.

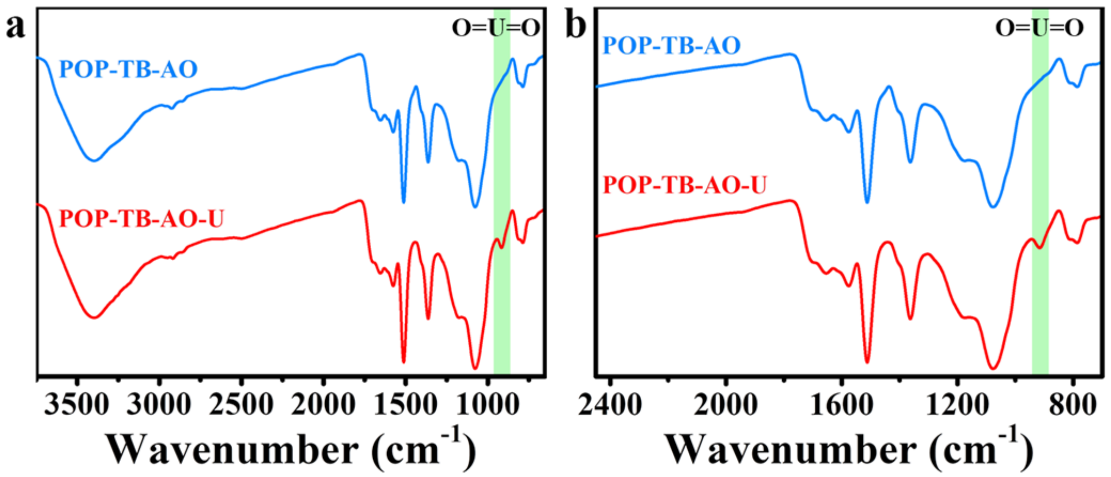

Supplementary Figure 21 | (a) FT-IR spectra of POP-TB-AO and POP-TB-AO-U. (b) Zoomed FT-IR spectra of POP-TB-AO and POP-TB-AO-U. The significant shift of the antisymmetric vibration of O=U=O in POP-TB-AO (919 cm^{-1}) in comparison with that in $UO_2(NO_3)_2{\cdot}6H_2O$ (~960 cm^{-1})[17] from the FT-IR studies further confirms that strong interactions exist between the UO_2^{2+} and POP-TB-AO.

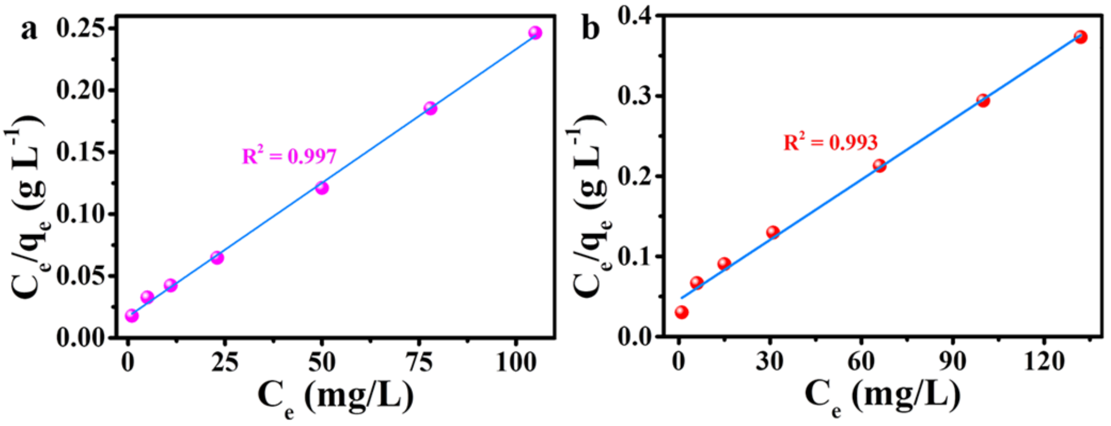

Supplementary Figure 22 | Linear regression by fitting the equilibrium adsorption data of TFPT-BTAN-AO (a) and POP-TB-AO (b) with Langmuir adsorption model. The adsorption data of TFPT-BTAN-AO and POP-TB-AO fit well with the Langmuir model as indicated by the high values of the correlation coefficients ($R^2 > 0.992$).

Supplementary Table 5 | UO_2^{2+} sorption performance of various sorbent materials.

Adsorbents	capacity (mg g^{-1})	Reference
$K_2MnSn_2S_6$(KMS-1)	380	17
Mesoporous Carbon Materials	97	18
Am-p(AN-c-MAc)	51.5	19
MOF-76	298	20
MIL-101-DETA	350	21
MIL-101(Cr)-triazole-COOH	304	22
S_x-LDH	330	23
FJSM-GAS-1	196	24
FJSM-SnS	338	25
COF-TpDb-AO	408	26
POP-TpDb-AO	355	27
POP-*o*NH_2-AO	530	27
Fe_3O_4@ZIF-8	523.5	28
GO-pDA-PEI	530.6	29
Hollow C-HCN-AO sphere	355.6	30
N, P, and S codoped graphene-like carbon nanosheets	294.16	31
Azine linked covalent organic framework	169	32
Conjugated microporous polymers (CMPAO)	251.9	33
o-GS-COF	144.2	34
TFPT-BTAN-AO	427	This work

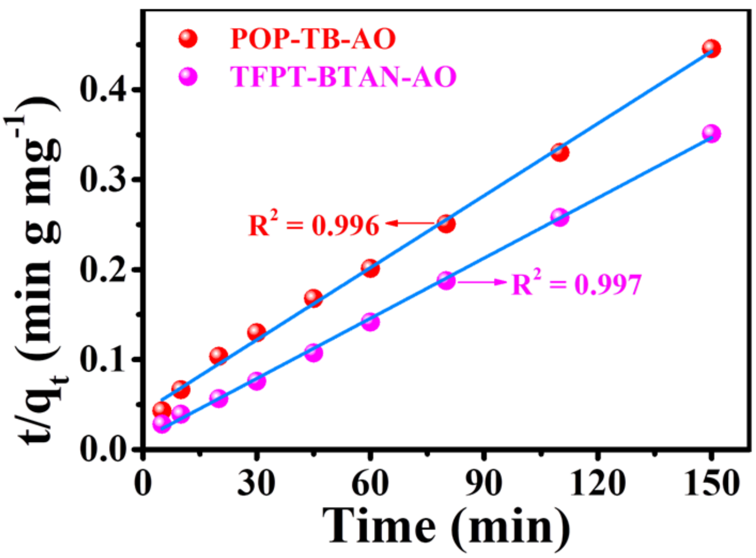

Supplementary Figure 23 | The pseudo-second-order kinetic plot for the adsorption UO_2^{2+} on TFPT-BTAN-AO and POP-TB-AO. The adsorption kinetic process fit well with the pseudo second-order kinetic model as indicated by the high values of the correlation coefficients ($R^2 > 0.995$). The pseudo second-order kinetic model expressed as equation (2):

$$\frac{t}{q_t} = \frac{1}{k_2 q_e^2} + \frac{t}{q_e} \tag{2}$$

Where k_2 (g mg^{-1} min^{-1}) is the pseudo second-order rate constant of adsorption, q_e and q_t are the adsorbed amount (mg g^{-1}) at equilibrium and at time t (min), respectively. The value of the adsorption rate constant k_2 was determined to be 0.0041 and 0.0017 g mg^{-1} min^{-1} for TFPT-BTAN-AO and POP-TB-AO, respectively, indicating that the COF-based adsorbent exhibits a higher adsorption rate in relation to its amorphous analog.

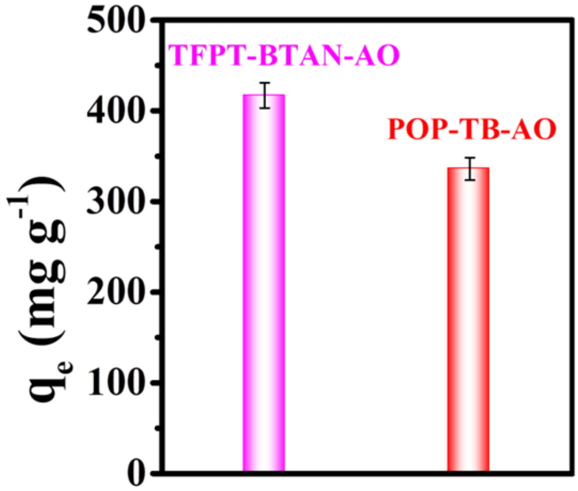

Supplementary Figure 24 | The UO_2^{2+} adsorption capacities of TFPT-BTAN-AO and POP-TB-AO from aqueous solution with an initial concentration of 19.9 ppm (200 mL), at pH = 4.0, and adsorbent material (5.0 mg). Error bars represent S.D. *n* =3 independent experiments.

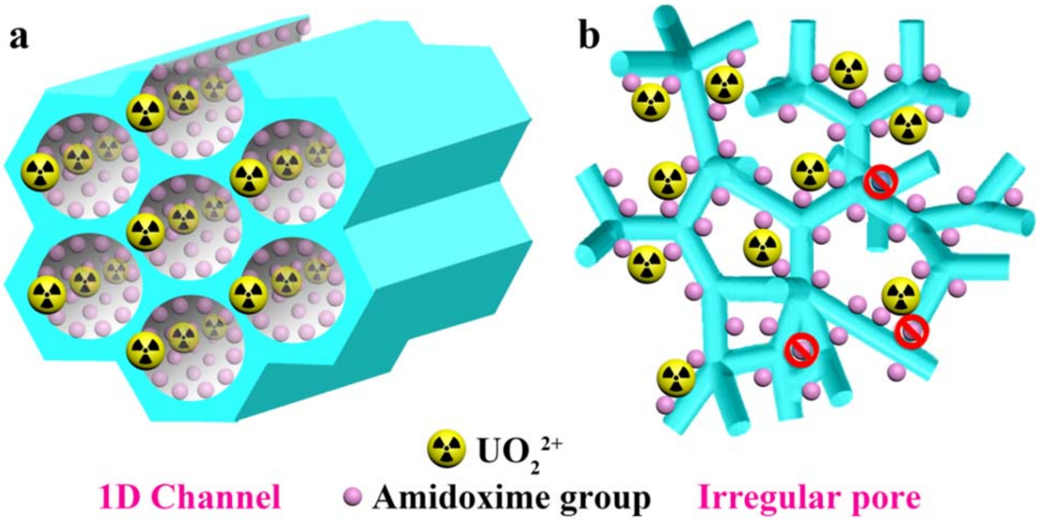

Supplementary Figure 25 | (A) Schematic representation of the chelation sites in TFPT-BTAN-AO to promote rapid diffusion of UO_2^{2+} throughout the framework. The hierarchical pore and 1D channel structure of TFPT-BTAN-AO results in the ability of the functionalized material to have unrestricted UO_2^{2+} entry into all chelating sites. (B) Functionalization of amorphous POP-TB-AO, illustrating clogging of narrow channels and necks. Blockage of the pores may impede UO_2^{2+} from entering the chelation site in POP-TB-AO.

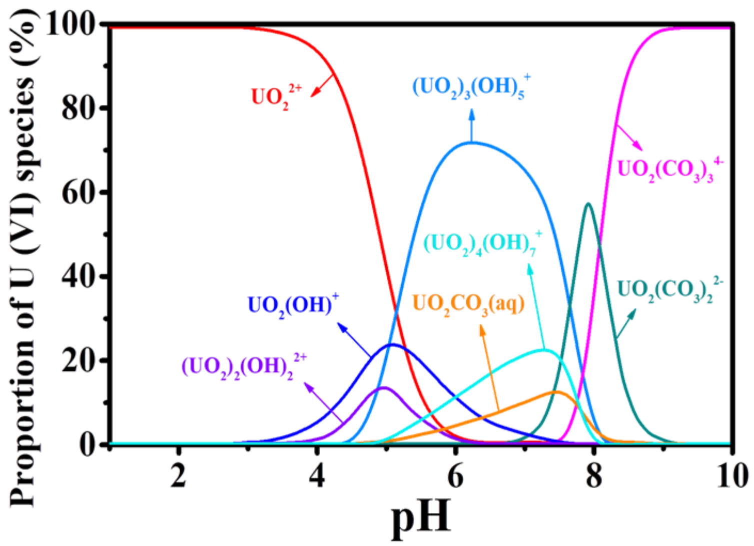

Supplementary Figure 26 | The relative distribution of U(VI) species in solution at various pH values (T = 293 K, $C_{U(VI)initial}$ = 7.5 × 10^{-5} mol/L).

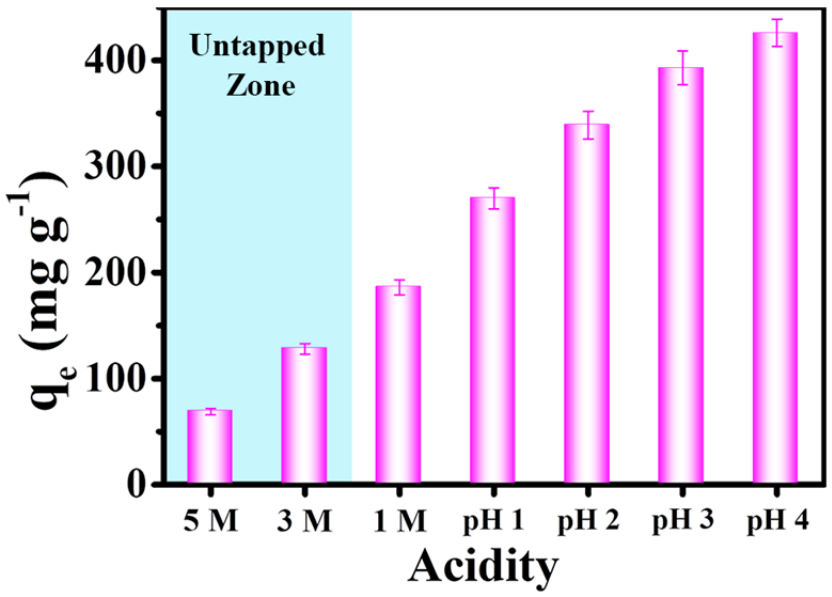

Supplementary Figure 27 | The UO_2^{2+} adsorption capacities of TFPT-BTAN-AO at different nitric acid concentrations and pH values, and compared with other reference materials. Since nuclear fuel reprocessing and wastewater are usually treated under highly acidic conditions, it is necessary to evaluate the effect of solution acidity on UO_2^{2+} extraction by TFPT-BTAN-AO. Error bars represent S.D. *n* =3 independent experiments.

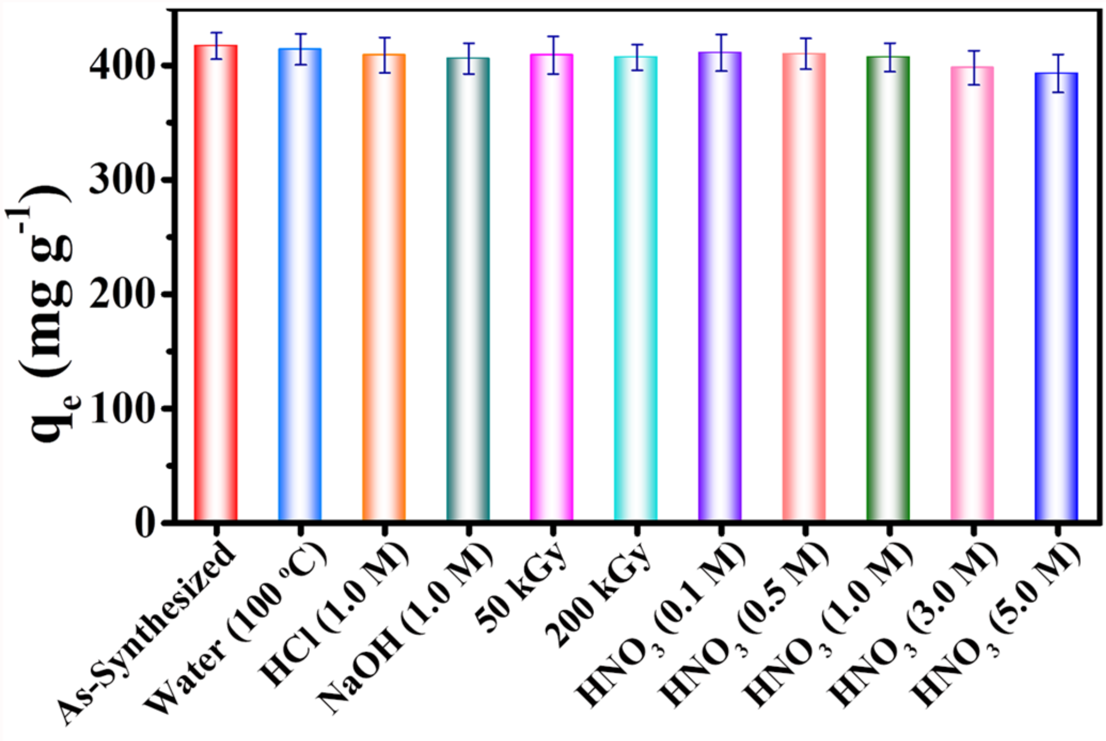

Supplementary Figure 28 | The adsorption performances of TFPT-BTAN-AO before and after treatment with water (100 °C), HCl (1.0 M), NaOH (1.0 M), γ-ray irradiation (50 kGy, 200 kGy), and HNO_3 (0.1-5.0 M). The results verified that the uranium extraction performance of TFPT-BTAN-AO was almost unchanged after treatment under various extreme conditions, indicating that TFPT-BTAN-AO has excellent stability and practical application potential. The UO_2^{2+} adsorption capacities of TFPT-BTAN-AO from aqueous solution with an initial concentration of 19.9 ppm (200 mL), at pH = 4.0, and adsorbent material (5.0 mg). Error bars represent S.D. *n* =3 independent experiments.

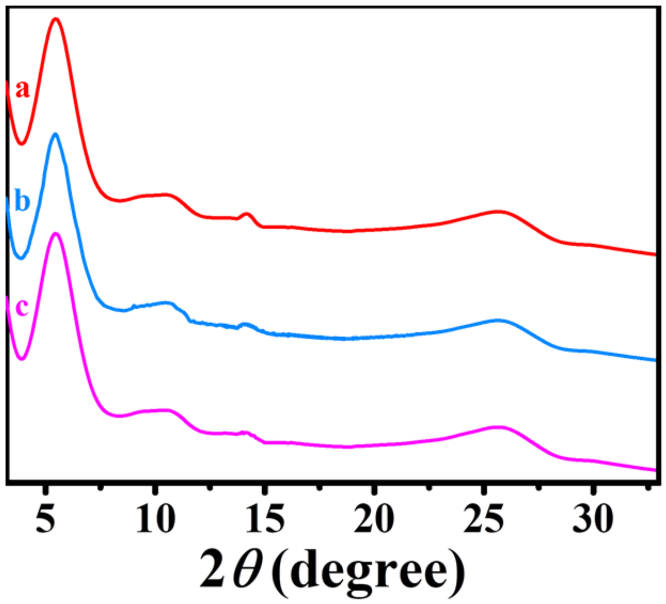

Supplementary Figure 29 | PXRD patterns of TFPT-BTAN-AO (a), TFPT-BTAN-AO after extraction of UO_2^{2+} (b), and TFPT-BTAN-AO after desorption of UO_2^{2+} by the Na_2CO_3 (1.0 mol L^{-1}) aqueous solution (c).

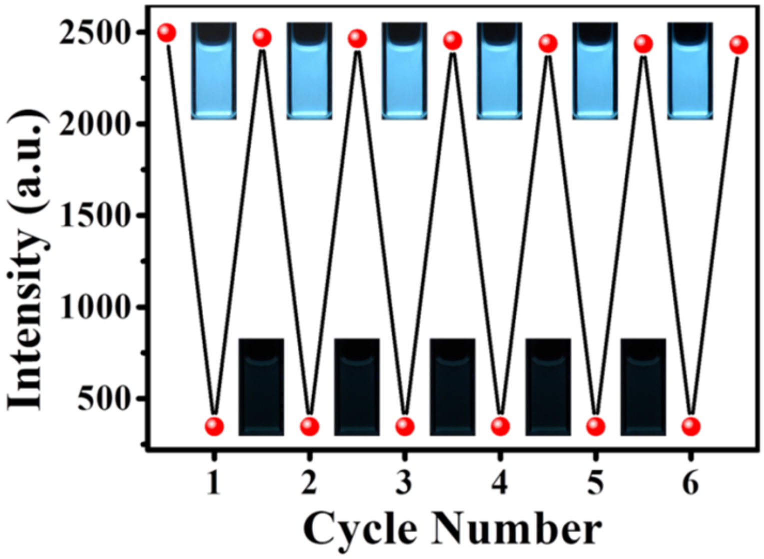

Supplementary Figure 30 | Recycle use of TFPT-BTAN-AO for selective detection and easy extraction of UO_2^{2+}. After treating with the Na_2CO_3 (1.0 mol L^{-1}) aqueous solution, TFPT-BTAN-AO was easily recovered and could be repeatedly used. Inset photos show the corresponding fluorescence emission change (under a 365 nm UV lamp) of TFPT-BTAN-AO after extraction and desorption of UO_2^{2+}.

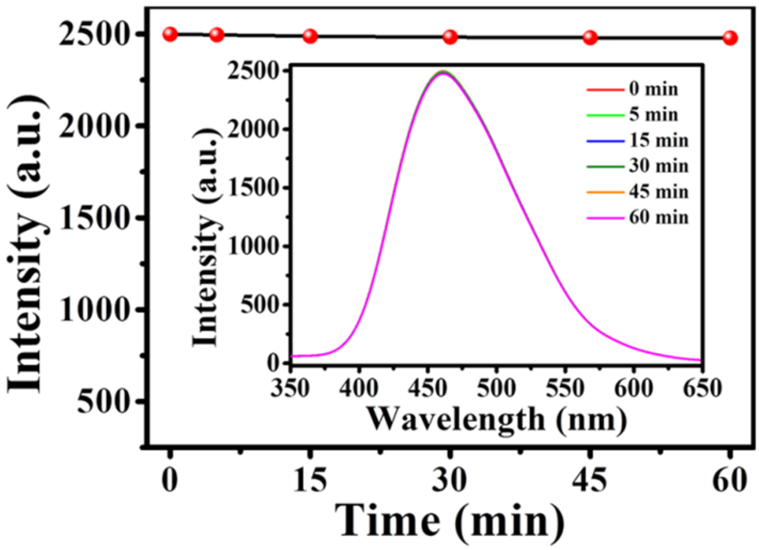

Supplementary Figure 31 | Time-dependent fluorescence intensity of TFPT-BTAN-AO tested within 60 minutes. The unchanged intensity indicates that TFPT-BTAN-AO does not show any photo-bleaching, and, the observed decrease in intensities in other cases is indeed induced by the addition of ions, such as UO_2^{2+}.

Liquid NMR Spectra

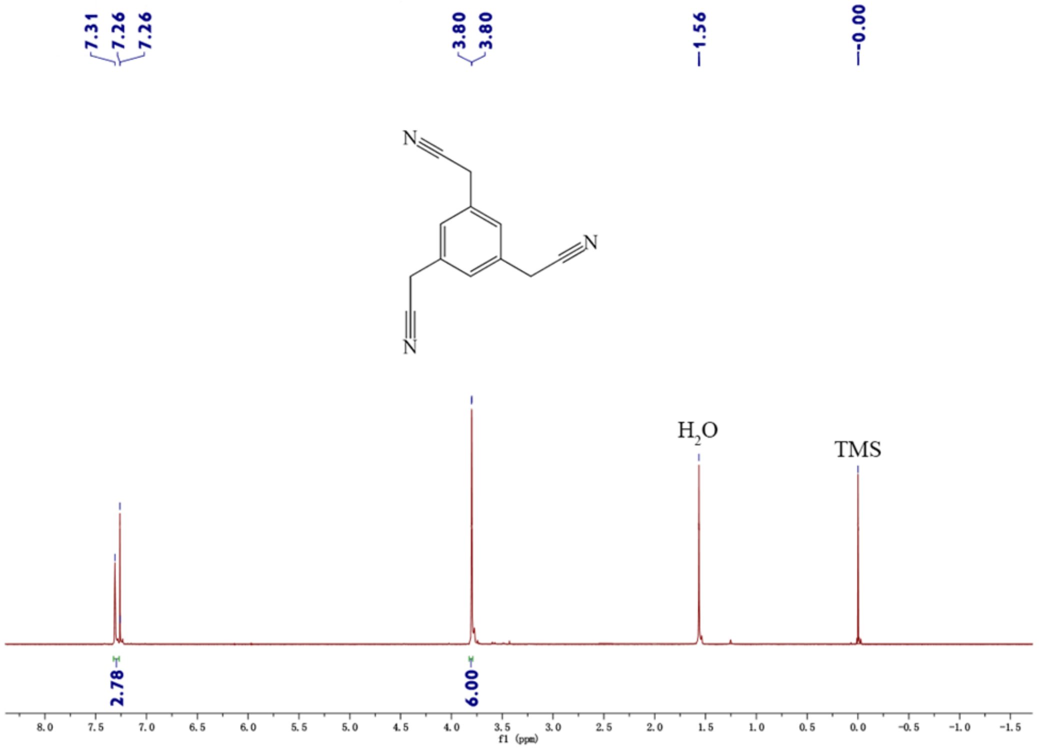

Supplementary Figure 32 | ^{1}H NMR spectra of 2,2',2''-(benzene-1,3,5-triyl)triacetonitrile (BTAN) in $CDCl_3$.

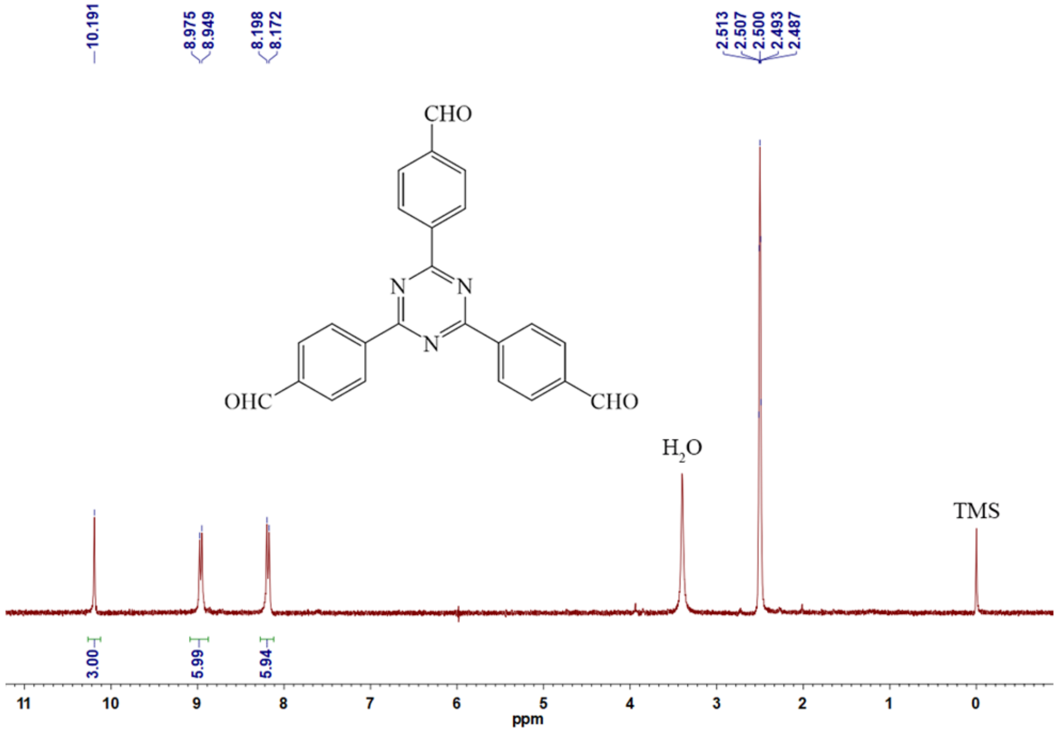

Supplementary Figure 33 | ^{1}H NMR spectra of 2,4,6-Tris(4-formylphenyl)-1,3,5-triazine (TFPT) in DMSO-d[6].

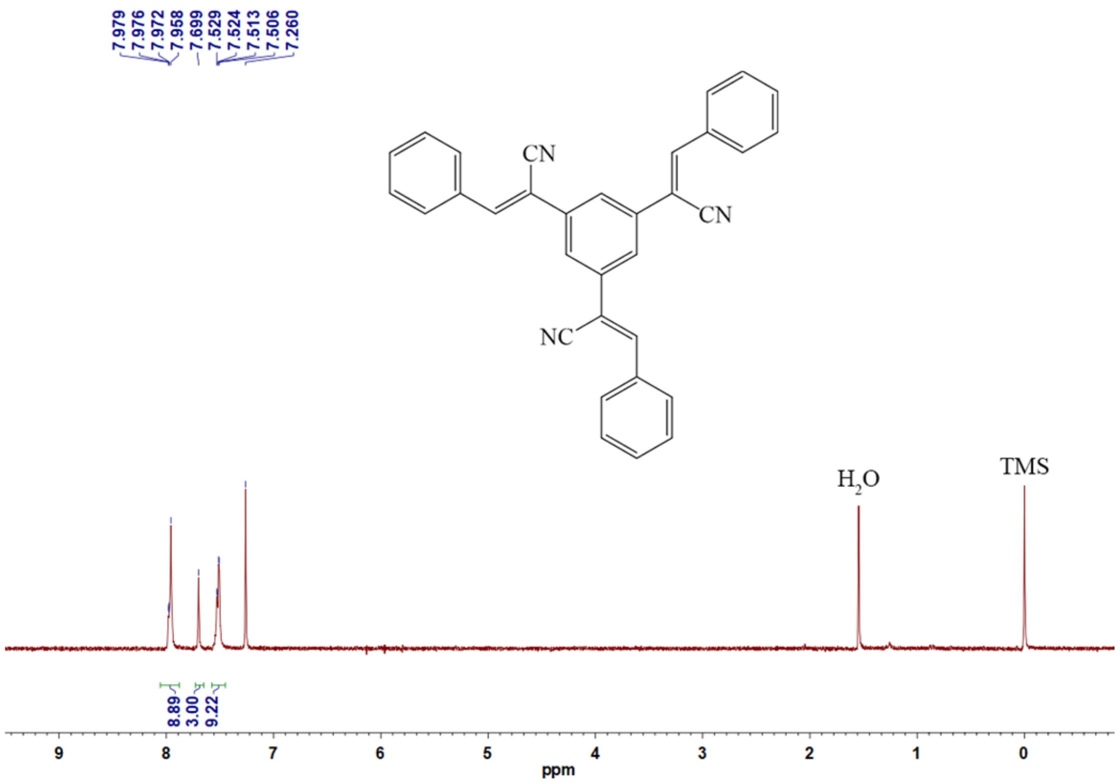

Supplementary Figure 34 | ^{1}H NMR spectra of Model Compound in $CDCl_3$.

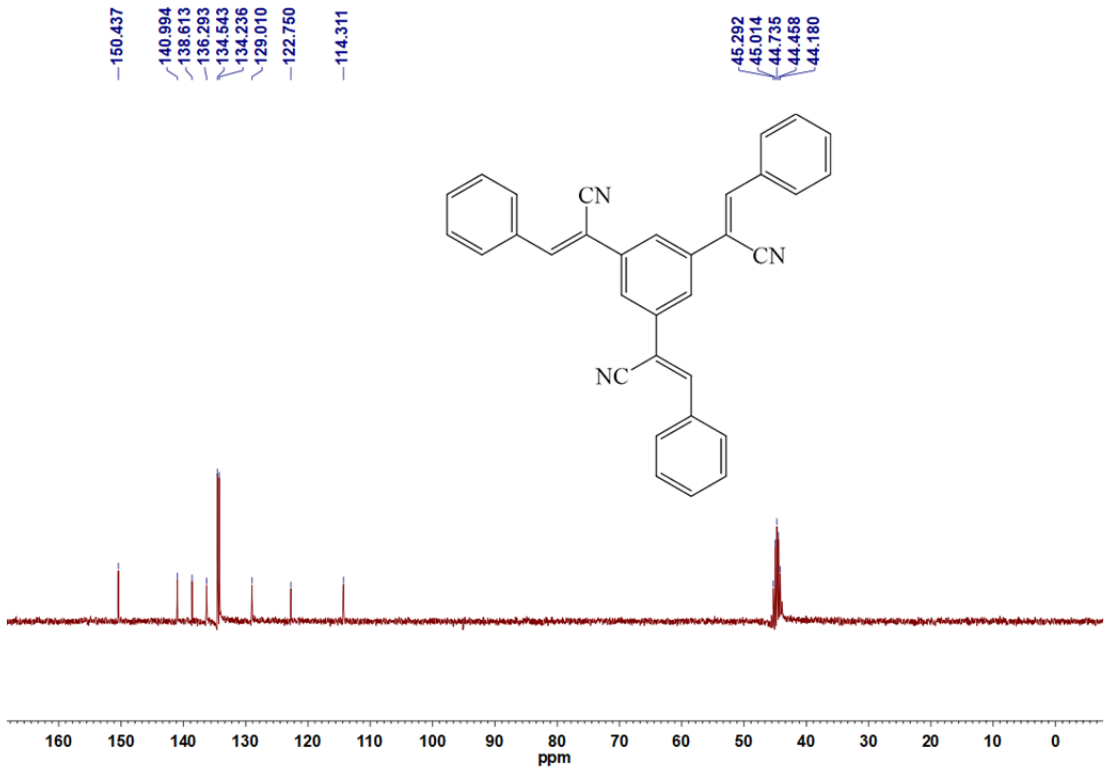

Supplementary Figure 35 | ^{13}C NMR spectra of Model Compound in DMSO-d^6.

Supplementary References

1 Yassin, A. *et al.* Structure-thermodynamic-property relationships in cyanovinyl-based microporous polymer networks for the future design of advanced carbon capture materials. *Adv. Funct. Mater.* **27**, 1700233 (2017).

2 Sekizkardes, A. K. *et al.* Highly selective CO_2 capture by triazine-based benzimidazole-linked polymers. *Macromolecules* **47**, 8328-8334 (2014).

3 Gadelle, F. *et al.* Removal of uranium(VI) from contaminated sediments by surfactants. *J. Environ. Qual.* **30**, 470-478 (2001).

4 Jarczewska, M. *et al.* Electrochemical uranyl cation biosensor with DNA oligonucleotides as receptor layer. *Bioelectrochemistry* **96**, 1-6 (2014).

5 Hosseini, M. S. *et al.* Synergistic flotation of U(VI)-alizarin complex with some diamines followed by spectrophotometric determination of U(VI) using 4,4′-diaminophenylmethane. *Anal. Chim. Acta* **559**, 181-185 (2006).

6 Dutta, R. K. & Kumar, A. Highly sensitive and selective method for detecting ultratrace levels of aqueous uranyl ions by strongly photoluminescent-responsive amine-modified cadmium sulfide quantum dots. *Anal. Chem.* **88**, 9071-9078 (2016).

7 Saha, A. *et al.* Micellar extraction assisted fluorometric determination of ultratrace amount of uranium in aqueous samples by novel diglycolamide-capped quantum dot nanosensor. *Sensor. Actuat. B: Chem.* **253**, 592-602 (2017).

8 Wen, J. *et al.* Aggregation-induced emission active tetraphenylethene-based sensor for uranyl ion detection. *J. Hazard. Mater.* **318**, 363-370 (2016).

9 Liu, W. *et al.* Highly sensitive and selective uranium detection in natural water systems using a luminescent mesoporous metal–organic framework equipped with abundant lewis basic sites: A combined batch, X-ray absorption spectroscopy, and first principles simulation investigation. *Environ. Sci. Technol.* **51**, 3911-3921 (2017).

10 Chandrasekaran, K. *et al.* Dispersive liquid–liquid micro extraction of uranium(vi) from groundwater and seawater samples and determination by inductively coupled plasma–optical emission spectrometry and flow injection–inductively coupled plasma mass spectrometry. *Anal. Methods* **3**, 2140-2147 (2011).

11 Ma, J. *et al.* Amidoximated fluorescent polymer based sensor for detection of trace uranyl ion in aqueous solution. *Talanta* **168**, 10-15 (2017).

12 Veyseh, S. & Niazi, A. A novel aeration-assisted homogenous liquid–liquid microextration for determination of thorium and uranium in water and hair samples by inductively coupled plasma-mass spectroscopy. *Talanta* **147**, 117-123 (2016).

13 Shamsipur, M. *et al.* One-step synthesis and characterization of highly luminescent nitrogen and phosphorus co-doped carbon dots and their application as highly selective and sensitive nanoprobes for low level detection of uranyl ion in hair and water samples and application to cellular imaging. *Sensor. Actuat. B: Chem.* **257**, 772-782 (2018).

14 Hua, M. *et al.* Highly selective and sensitive determination of uranyl ion by the probe of CdTe quantum dot with a specific size. *Talanta* **190**, 278-283 (2018).

15 Zhang, Z. *et al.* 3,4-Hydroxypyridinone-modified carbon quantum dot as a highly sensitive and selective fluorescent probe for the rapid detection of uranyl ions. *Environ. Sci. Nano* **21**, 649-657 (2019).

16 Cui, W.-R. *et al.* Covalent organic framework nanosheet-based ultrasensitive and selective

colorimetric sensor for trace Hg^{2+} detection. *ACS Sustainable Chem. Eng.* **7**, 9408-9415 (2019).

17 Manos, M. J. & Kanatzidis, M. G. Layered metal sulfides capture uranium from seawater. *J. Am. Chem. Soc.* **134**, 16441-16446 (2012).

18 Carboni, M. *et al.* Uranium sorption with functionalized mesoporous carbon materials. *Ind. Eng. Chem. Res.* **52**, 15187-15197 (2013).

19 Sahiner, N. *et al.* Highly porous acrylonitrile-based submicron particles for UO_2^{2+} absorption in an immunosensor assay. *ACS Appl. Mater. Interfaces* **4**, 163-170 (2012).

20 Yang, W. *et al.* MOF-76: from a luminescent probe to highly efficient U(VI) sorption material. *Chem. Commun. (Camb.)* **49**, 10415-10417 (2013).

21 Bai, Z.-Q. *et al.* Introduction of amino groups into acid-resistant MOFs for enhanced U(vi) sorption. *J. Mater. Chem. A* **3**, 525-534 (2015).

22 Li, L. *et al.* A combined experimental and theoretical study on the extraction of uranium by amino-derived metal–organic frameworks through post-synthetic strategy. *ACS Appl. Mater. Interfaces* **8**, 31032-31041 (2016).

23 Ma, S. *et al.* Efficient uranium capture by polysulfide/layered double hydroxide composites. *J. Am. Chem. Soc.* **137**, 3670-3677 (2015).

24 Feng, M.-L. *et al.* Efficient removal of $[UO_2]^{2+}$, Cs^+, and Sr^{2+} ions by radiation-resistant gallium thioantimonates. *J. Am. Chem. Soc.* **140**, 11133-11140 (2018).

25 Feng, M.-L. *et al.* Efficient removal and recovery of uranium by a layered organic–inorganic hybrid thiostannate. *J. Am. Chem. Soc.* **138**, 12578-12585 (2016).

26 Sun, Q. *et al.* Covalent organic frameworks as a decorating platform for utilization and affinity enhancement of chelating sites for radionuclide sequestration. *Adv. Mater.* **30**, 1705479 (2018).

27 Sun, Q. *et al.* Bio-inspired nano-traps for uranium extraction from seawater and recovery from nuclear waste. *Nat. Commun.* **9**, 1644 (2018).

28 Min, X. *et al.* Fe_3O_4@ZIF-8: a magnetic nanocomposite for highly efficient UO_2^{2+} adsorption and selective UO_2^{2+}/Ln^{3+} separation. *Chem. Commun. (Camb.)* **53**, 4199-4202 (2017).

29 Li, S. *et al.* Graphene oxide based dopamine mussel-like cross-linked polyethylene imine nanocomposite coating with enhanced hexavalent uranium adsorption. *J. Mater. Chem. A* (2019).

30 Liu, X. *et al.* Amidoxime-functionalized hollow carbon spheres for efficient removal of uranium from wastewater. *ACS Sustainable Chem. Eng.* **7**, 10800-10807 (2019).

31 Chen, Z. *et al.* N, P, and S Codoped graphene-like carbon nanosheets for ultrafast uranium (VI) capture with high capacity. *Adv. Sci.* **5**, 1800235 (2018).

32 Li, X. *et al.* Solvent- and catalyst-free synthesis of an azine-linked covalent organic framework and the induced tautomerization in the adsorption of U(vi) and Hg(ii). *Green Chem.* (2019).

33 Xu, M. *et al.* Highly fluorescent conjugated microporous polymers for concurrent adsorption and detection of uranium. *J. Mater. Chem. A* **7**, 11214-11222 (2019).

34 Wen, R. *et al.* Graphene-synergized 2D covalent organic framework for adsorption: a mutual promotion strategy to achieve stabilization and functionalization simultaneously. *J. Hazard. Mater.* **358**, 273-285 (2018).
